# *Mid1/Mid2* expression in craniofacial development and a literature review of X‐linked opitz syndrome

**DOI:** 10.1002/mgg3.183

**Published:** 2015-12-12

**Authors:** Bijun Li, Tianhong Zhou, Yi Zou

**Affiliations:** ^1^ Department of BiologyJinan UniversityGuangzhouChina

**Keywords:** Craniofacial development, functional redundancy, *MID1*, *MID2*, midline defects, X‐linked Opitz syndrome

## Abstract

**Background:**

Opitz syndrome (OS) is a genetic disorder that affects mainly the development of midline structures, including the craniofacial region, embryonic heart, and urogenital system. The manifestations of X‐linked OS are believed to be results of a malfunctioned gene, MID1, whose product has been shown to have ubiquitin E3 ligase activity and regulate the turnover of microtubular protein phosphatase 2Ac. MID2, a homolog of MID1, shares high structural and functional similarities with MID1. Identification of a missense mutation in MID2 in an Indian family causing overlapping phenotypes with OS provided the first evidence that MID2 might be involved in similar pathogenesis.

**Methods:**

The clinic features and the genetic findings of all reported X‐linked OS were collectively summarized in this research. Real‐time RT‐PCR and *in situ* hybridization were used in the expression studies of *Mid1/Mid2* in mouse embryos.

**Results:**

Up‐to‐date, 88 different mutations have been identified in MID1 and most mutations occurred on the conserved amino acids of MID1 and MID2. Expression studies using real‐time RT‐PCR implicated a tendency of a mutually repressive expression pattern between Mid1 and Mid2 in mouse embryos. Further investigations using in situ hybridization revealed strong expressions of Mid1 and Mid2 in the epithelium of approaching facial prominences and downregulated expressions after fusion in mouse embryos.

**Conclusions:**

Our results support the hypothesis of functional redundancy of Mid1/Mid2 and their potential roles in regulating tissue remodelling in early development.

## Introduction

Opitz syndrome (OS) was first reported by Opitz as two different syndromes, the BBB syndrome and the G syndrome, which were found, in fact, to be the same condition. OS represents a genetic disorder of the primary midline developmental field and is characterized by a variable array of features that includes facial abnormalities, urogenital defects, and congenital heart defects (Cox et al. 2000).

The disorder is genetically heterogeneous with both X‐linked and autosomal loci, Xp22.3 and 22q11.2, respectively. Deletion of the overlapping region on 22q11.2 has also been found in other syndromes with shared clinical features, such as the DiGeorge syndrome (DGS), velo‐cardio‐facial syndrome (VCFS), and conotruncal anomaly face syndrome (CAFS) (Scambler 2000).

Since the implication of *MID1* (OMIM# 300552) as the causative gene for X‐linked OS (XLOS), over 88 different mutations have been found in sporadic and familial OS cases (Quaderi et al. 1997; Gaudenz et al. 1998; Schweiger et al. 1999; Cox et al. 2000; De Falco et al. 2003; Winter et al. 2003; Pinson et al. 2004; So et al. 2005; Cho et al. 2006; Mnayer et al. 2006; Shaw et al. 2006; Ferrentino et al. 2007; Fontanella et al. 2008; Hsieh et al. 2008; Hu et al. 2012; Hüning et al. 2013; Migliore et al. 2013; Ji et al. 2014). *MID1* mutations scattered throughout the gene, including mutations in noncoding regions that affect the expression of *MID1* may also be present in some cases (Cox et al. 2000). The developmental expression profile of *MID1* is also consistent with this causative role with the highest levels of expression found in the affected tissues (Dal Zotto et al. 1998; Richman et al. 2002). However, the identification of *MID1* mutations in <50% of X‐linked OS and the variability in clinical phenotypes of patients carrying the same *MID1* mutation even within the same family, strongly suggest the existing of other OS‐related genes.

Retrieval and assembly of full length cDNA revealed an ORF of 2055 bp, *MID2* (OMIM#300204), encoding a 685‐amino‐acid protein with 83% amino acid similarity (76% identity) with MID1. Like *MID1*,* MID2* maps to the X chromosome but to the long arm at band Xq22 (Xp22 for *MID1*). Analysis of gene composition and order on the X‐chromosome indicated that Xp22 and Xq22, the loci for *MID1* and *MID2*, respectively, may have resulted from an ancient intrachromosomal duplication (Perry et al. 1998; Buchner et al. 1999).

MID1 and MID2 are both RBCC (RING‐finger, B‐boxes and Coiled‐coil) proteins, which are characterized by their ability to act as scaffolds with multiple protein–protein interactions. Existing data suggest that MID1 and MID2 may overlap in their function: both MID1 and MID2 are found distributed along microtubules in all cell types examined and this localization is important for their function (Cainarca et al. 1999); homo‐ or hetero‐dimerization is a prerequisite for their microtubule‐association (Short et al. 2002); both MID1 and MID2 interact with alpha4 and ubiquitylate alpha4′s downstream target, protein phosphatase 2A (PP2A), which in turn regulates MID1 and MID2 turnover on microtubules (Liu et al. 2001; Short et al. 2002; Aranda‐Orgilles et al. 2008; McConnell et al. 2010; LeNoue‐Newton et al. 2011; Latta and Golding 2012; Du et al. 2014); both have potential E3 ubiquitin ligase activity (Trockenbacher et al. 2001; Han et al. 2011); disrupted function of MID1/MID2 resulted in Xenopus neural tube defects due to disorganization of microtubules (Suzuki et al. 2010); and functional redundancy of MID1/MID2 has also been displayed in avian left–right determination during early development in chick embryo (Granata et al. 2005). Furthermore, duplication of Xq22.3, a region including *hMID2*, has been identified as a new locus for FG syndrome, which shares many clinical manifestations with OS (Jehee et al. 2005). Coincidently, identification of a duplication of Xp22.3 (a region including *hMID1*) with a terminal deletion of 4p in a patient diagnosed with OS and Wolf‐Hirschhorn syndrome indicated the gene dosage of *MID1/MID2* may also be critical in regulating early development (So et al. 2008). Recently, a missense mutation (c.1040G>A, p.Arg347Gln) in *MID2* was identified in a large Indian family and was shown to cause X‐linked intellectual disability/developmental delay and minor facial changes, including short philtrum, large ears, and a squint. All these defects have also been reported in XLOS patients. It was the first time that a disease causative mutation in *MID2* was identified, providing strong evidence to support a role for *MID2* in regulating similar processes during early development (Geetha et al. 2013). However, very little research has been carried out with regard to the expression pattern and cellular function of *MID2*.

A thorough literature review of all the reported X‐linked OS was presented in this work and craniofacial abnormalities, including hypertelorism and cleft lip/palate, remained to be the most prominent facial defects (presented in 99% and 47% OS cases, respectively), consistent with previous report (Fontanella et al. 2008). Hypospadias was seen in 80% cases, in line with previous studies (So et al. 2005; Fontanella et al. 2008). In considering the developmental processes that form the various structures affected in OS patients, including the midface, external genitalia and heart, defects in tissue remodelling, and fusion of the embryonic ventral midline were likely to be essential for OS pathogenesis. Therefore, the expressions of *Mid1* and *Mid2* were examined and compared to the relevant structures in mouse embryos by real‐time RT‐PCR in this study. Like *Mid1*,* Mid2* was ubiquitously expressed in embryonic mouse tissues from E9.5 days postcoitum (dpc) throughout to E13.5 dpc, with an overall lower expression level than that of *Mid1*, except in the developing heart. Complementary expression patterns were observed for *Mid1* and *Mid2*. The peak expression of *Mid2* appeared around E11.5 dpc in the craniofacial region, central nervous system (CNS), and the embryonic heart, while the level of *Mid1* dropped dramatically around E11.5 dpc. Detailed expressions of both *Mid1* and *Mid2* were further investigated in craniofacial regions in these mouse embryos by in situ hybridization and high expressions were revealed for both *Mid1* and *Mid2* in the epithelium and in the adjacent mesenchyme of approaching facial prominences. Our results in this study support the hypothesis of functional redundancy of Mid1/Mid2 and potential roles for MID1/MID2 in regulating tissue remodelling during OS pathogenesis.

## Materials and Methods

### Mouse embryo collection and section

KM mice were purchased from Laboratory Animal Center of Sun Yat‐Sen University. Mice were housed in an environmentally controlled facility and allowed free access to food and water. Timed matings of mice were conducted by placing females with fertile males, and embryos were staged from the presence of a vaginal plug representing 0.5 dpc. Embryos were removed from pregnant mice at different developmental stages after death by cervical dislocation. Embryos were processed either for in situ hybridization or reverse transcription quantitative real‐time PCR. For in situ hybridization, embryos were fixed with 4% paraformaldehyde in PBS overnight at 4°C. The embryos were then washed three times in DEPC‐treated PBS, dehydrated through graded ethanol: 25, 50, 75, 95, and 100%, and embedded in paraffin. Whole embryos were embedded in sagittal plane. Heads of embryos were removed and embedded into the frontal and transverse planes across the midfacial region. Sections were made, 8‐*μ*m thick. Sagittal, transverse, and frontal sections as indicated by horizontal lines in schematic diagram were collected. The sections were placed onto 3'‐aminopropyl‐triethoxysilane‐coated glass slides (Beijing Dingguo Changsheng, Beijing, China) and baked at 42°C overnight. The slides were stored at room temperature. For reverse transcription quantitative real‐time PCR, craniofacial regions, CNSs and heart tubes were dissected from embryos. The total RNAs were then purified using the TRIzol method (TRIzol reagent; Invitrogen, Carlsbad, CA, USA).

### RNA in situ hybridiztion

Oligonucleotide probes were designed to target the *Mid1* and *Mid2* gene. The *Mid1* probes encompass nt 1674‐1711 of the *Mid1* coding sequence (CCDS41215.1). For the *Mid2* gene, the probes corresponding to nt 1632–1676 of the *Mid2* coding sequence (CCDS41151.1) were selected. Both digoxygenin‐labeled sense and antisense probes were synthesized by Sangon Biotech, Shanghai, China.

Mouse embryo tissue sections were deparaffinized and hydrated through 100, 95, 75, and 50, 25% ethanol, and DEPC‐treated distilled water. RNA in situ hybridization experiments were preformed according to the manufacturer's instructions (In Situ Hybridization Kit; Beijing Dingguo Changsheng). Sections were postfixed with 4% paraformaldehyde in PBS for 15 min, washed with DEPC‐treated PBS, and bleached with 0.5% H_2_O_2_ in methanol for 30 min. Sections were then washed three times with DEPC‐treated distilled water. Sections were treated with proteinase K for 20 min, washed with DEPC‐treated PBS, and postfixed with 4% paraformaldehyde in PBS for 15 min. After 2 h of prehybridization with prehybridization solution at 42°C, adjacent sections were hybridized with different digoxigenin‐labeled oligonucleotide probes. In order to localize *Mid1* transcript in embryo sections, in situ hybridization was performed overnight at 53°C in hybridization solution containing antisense probe (0.25 ng/*μ*L). As for localization of *Mid2* transcript, the probe was diluted in hybridization solution to the optimal concentration (0.05 ng/*μ*L), and added to each section. The sections were then covered with slips and incubated overnight at 51°C in a sealed, humidified RNase‐free box. The next day, hybridized sections were wished twice with 2 × SSC at 53°C (*Mid1*)/51°C (*Mid2*) for 20 min each. The wash was repeated twice with 0.5 × SSC and twice with 0.2 × SSC. Slides were equilibrated in PBS at room temperature for 15 min with rocking. Blocking solution was added to sections and incubated at 37°C for 30 min. The sections were then incubated with rabbit anti‐digoxygenin antibody at 37°C for 1 h, followed by four times wishing with PBS for 15 min each. AP‐conjugated anti‐rabbit secondary antibody was added to the sections and incubated at 37°C for 1 h. The sections were again washed with PBS twice for 15 min each, twice with TBS1 (0.1% Tween 20, pH 8.0) for 15 min each and twice with TBS2 (0.1% Tween 20, pH9.5) for 15 min each. Hybridization signals were detected by exposing the sections to the substrate for AP, nitroblue tetrazolium–5‐bromo‐4‐chloro‐3‐inodoyl phosphate (NBT‐BCIP). The color reaction was stopped by extensive washing with distilled water. Finally, the sections were wished with 95% ethanol, air‐dried, and photographed, using a Nikon TE2000‐S, Tokyo, Japan inverted microscope with Olympus LG‐PS2 light source. In all the experiments performed, no specific signal was detected with the digoxygenin‐labeled sense probe.

### Reverse transcription quantitative real‐time PCR

Total RNAs were reverse transcribed using oligo‐dT (18) primer (Sangon Biotech) and Moloney murine leukemia virus (M‐MLV) reverse transcriptase (TOYOBO, Osaka, Japan) at 42°C for 1 h. Reaction was stopped at 95°C for 5 min. The cDNA was quantified using the SYBR green method (SYBR Premix Ex Taq; TaKaRa, Dalian, China) and Applied Biosystems, Foster City, CA, USA 7300 Real‐Time PCR System. PCR reactions were set up using specific primers to amplify *Mid1* and *Mid2*. *Mid1* cDNA was amplified using the primers 5'‐GGTCACCAGCGGAAGCACA‐3' and 5'‐CAGACACTTGTT CCACACGGTG‐3'. *Mid2* cDNA was amplified using the primers 5'‐TACTGGGAGGTGGTCATG‐3' and 5'‐GAGTTAGCTGGGTCATAGA‐3'. Reaction conditions were 30 cycles of 30 sec at 98°C, 30 sec at 60°C, and 30 sec at 72°C. The input cDNA was normalized for PCR by using primers for *Gapdh* 5'‐CAAAATGGTGAAGGTCGG‐3' and 5'‐AGGTCAATGAAGGGGTCG‐3'.

## Results

### Literature review of OS

Clinical and other information of all reported X‐linked Opitz syndrome cases were reviewed from the literature. A total of 184 OS cases and their families were included in the clinical study. Pathogenic mutations in *MID1* gene have been verified in these OS patients by sequencing the original reports. We selected the OS index cases with detailed clinical description, that is, description of oral cavity abnormalities, brain abnormalities, and congenital cardiovascular defects, as the total number of informative cases and excluded those that were not clearly stated. For instance, as to hypertelorism, anteverted nares, and ear abnormalities, we marked as “+” or “−” for positive or negative when described in the original literature, while were left blank if not stated and excluded from statistics. Other major structural defects such as laryngotracheal anormalities, cardiovascular defects, hypospadias, and anal defects were considered negative if not addressed and included in our statistics. Negative reports of brain abnormalities were valued only if MRI was involved in diagnostics and were otherwise not included in our calculation.

We summarized 12 most common clinical features of these 184 XLOS and the relevant genetic defects (Table S1). The clinical features were less well described by Ferrentino et al. (2007) and Fontanella et al. (2008), and some structural abnormalities, such as laryngotracheal abnormalities, which could be companied with or without functional changes, were collectively addressed. Therefore, these cases were underlined and grouped separately as group b. Facial anomalies are the most prominent feature of OS, including hypertelorism, prominent forehead, widow's peak, broad and/or high nasal bridge, anteverted nares, broad and/or grooved nasal tip, flat philtrum, and ear abnormalities (Table S2).

Hypertelorism was found almost in every affected male individual except for just one case in group b and affected most female patients (Table 1). Ear anomalies affected 78% XLOS cases that were clearly addressed and were usually manifested as low‐set and posteriorly rotated ears. A few other XLOS had mild glue ear and hearing loss, which were included in ear anomalies in this review. Anteverted nares were found in 68% cases that were clearly addressed. Due to the affected development of branchial arch on the midline, cleft lip with or without cleft palate was another commonly seen craniofacial defect, occurred in almost half of XLOS, 49.4% cases in group a and 40% cases in group b, respectively. Other oral cavity abnormalities, such as highly arched palate, micrognathia, hypodontia, and abnormal uvula, have been found in sporadic individuals. Laryngotracheal abnormalities such as, laryngo‐esophageal cleft, fistula, and stenosis were also consequences of defective branchial arch development and affected almost half of XLOS. Some of these structural abnormalities were companied by dysphagia, aspiration, and gastroesophageal reflux while these problems could also present without any noticeable structural change. Therefore, we grouped the OS cases (group b, underlined) reported by Ferrentino et al. (2007) and Fontanella et al. (2008) since the structural changes and functional defects were not addressed separately. Other affected midline structures include CNS, cardiovascular system, and urogenital system. Hypospadias was the second most common feature and identified in nearly 80% male patients. Intellectual disability and cardiovascular defects have been reported in over 20% XLOS while the severities of these symptoms vary dramatically amongst different individuals (Tables S3 and S4).

**Table 1:** Incidence of the manifestations in X‐linked OS patients with identified *MID1* gene mutations

	Males^a^		Males^b^		Females^a^^,^ b	
Hypertelorism	82/82	100.0%	27/28	96.4%	38/42	90.5%
Anteverted nares	17/25	68.0%	–	–	1/42	2.4%
Cleft lip and/or palate	42/85	49.4%	14/35	40.0%	0/42	0.0%
Ear anomalies	32/41	78.0%	–	–	0/42	0.0%
Laryngotracheal abnormalities	40/85	47.0%	16/35	44.7%	1/42	2.4%
Dysphagia/aspiration/gastroesophageal reflux	31/85	36.4%			2/42	4.8%
Intellectual disability/MR/Dev delay	28/85	32.9%	5/35	14.3%	0/42	0.0%
Brain abnormalities	18/35	51.4%	2/6	33.3%	0/42	0.0%
Cardiovascular defects	20/85	23.5%	7/35	20.0%	0/42	0.0%
Hypospadias	68/85	80.0%	27/35	77.1%	0/42	0.0%
Anal defects	18/85	21.1%	4/35	11.4%	0/42	0.0%

a Quaderi et al. (1997); Gaudenz et al. (1998); Schweiger et al. (1999); Cox et al. (2000); De Falco et al. (2003); Winter et al. (2003); Pinson et al. (2004); So et al. (2005); Cho et al. (2006); Mnayer et al. (2006); Shaw et al. (2006); Hsieh et al. (2008); Hu et al. (2012); Hüning et al. (2013); Migliore et al. (2013); Ji et al. (2014).

b Ferrentino et al. (2007); Fontanella et al. (2008).

### Genetic defects of XLOS

**Figure 1:**
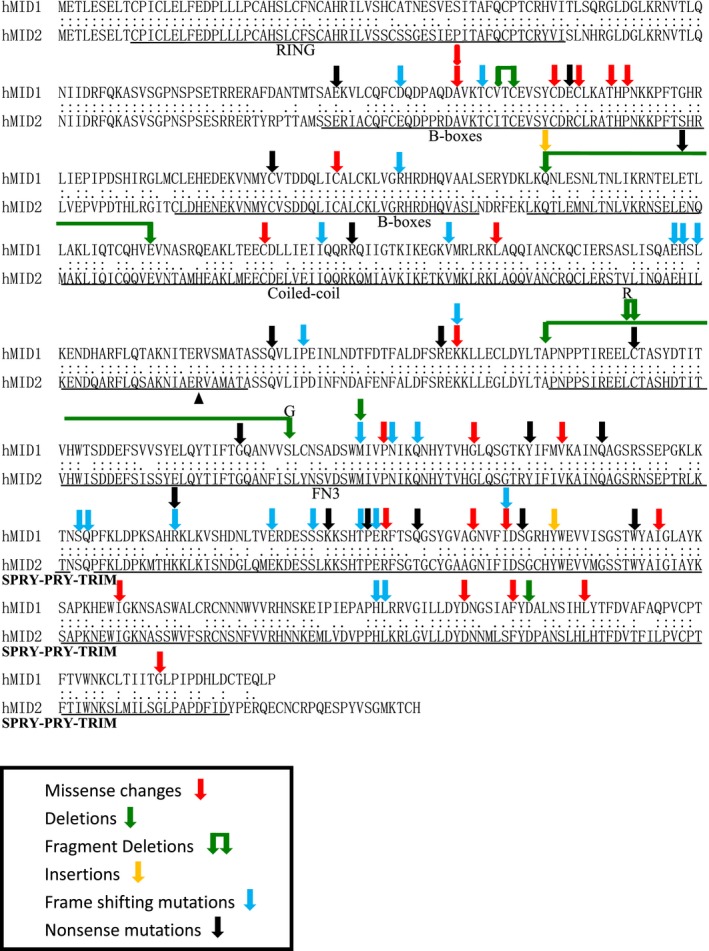
The genetic defects of X‐linked OS (XLOS). The genetic defects identified in all reported XLOS and their impacts on proteins were displayed on the protein sequence alignment of hMID1 and hMID2 (splice errors with unknown consequences on protein structures were not included). Missense mutations, deletions, insertions, frameshift mutations, and nonsense mutations were indicated with arrows of different colors on the corresponding amino acids on the sequence. The only disease‐causing mutation of *MID2* identified so far was also indicated with a black arrowhead on the corresponding amino acid of MID2 sequence.

The genetic defects of these 184 XLOS cases, including 53 females, have been summarized in Table 1. A total of 88 different mutations in *MID1* gene have been identified in these XLOS cases reviewed, including missense, nonsense mutations, in frame/frame shift insertions or deletions and splice errors. Four different mutations, p.Arg277X, p.Arg495X, p.Ser483LysfsX6, and the entire deletion of *MID1*, reoccurred in unrelated individuals. The mutations were scattered throughout the entire *MID1* gene but the RING domain. All mutation were found within the conserved sequences of MID1 (RefSeq# NP 000372.1) and MID2 (RefSeq# NP 438112.2) except two truncation mutations (p.Glu144X and p.Gln525X), occurred on the nonconserved amino acids of MID1 and MID2. No correlation has been found between the type/location of the mutation and the clinical features, as addressed by other groups (Fontanella et al. 2008). An Indian family with X‐linked intellectual disability caused by p.Arg347Gln missense mutation in MID2, indicated with an arrowhead in Figure 1, was recently reported. It was the first time that a disease causative mutation was assigned to *MID2* and this mutation also occurred on the conserved amino acids of MID1 and MID2.

### The expressions of *Mid1* and *Mid2* in developing mouse embryo

**Figure 2:**
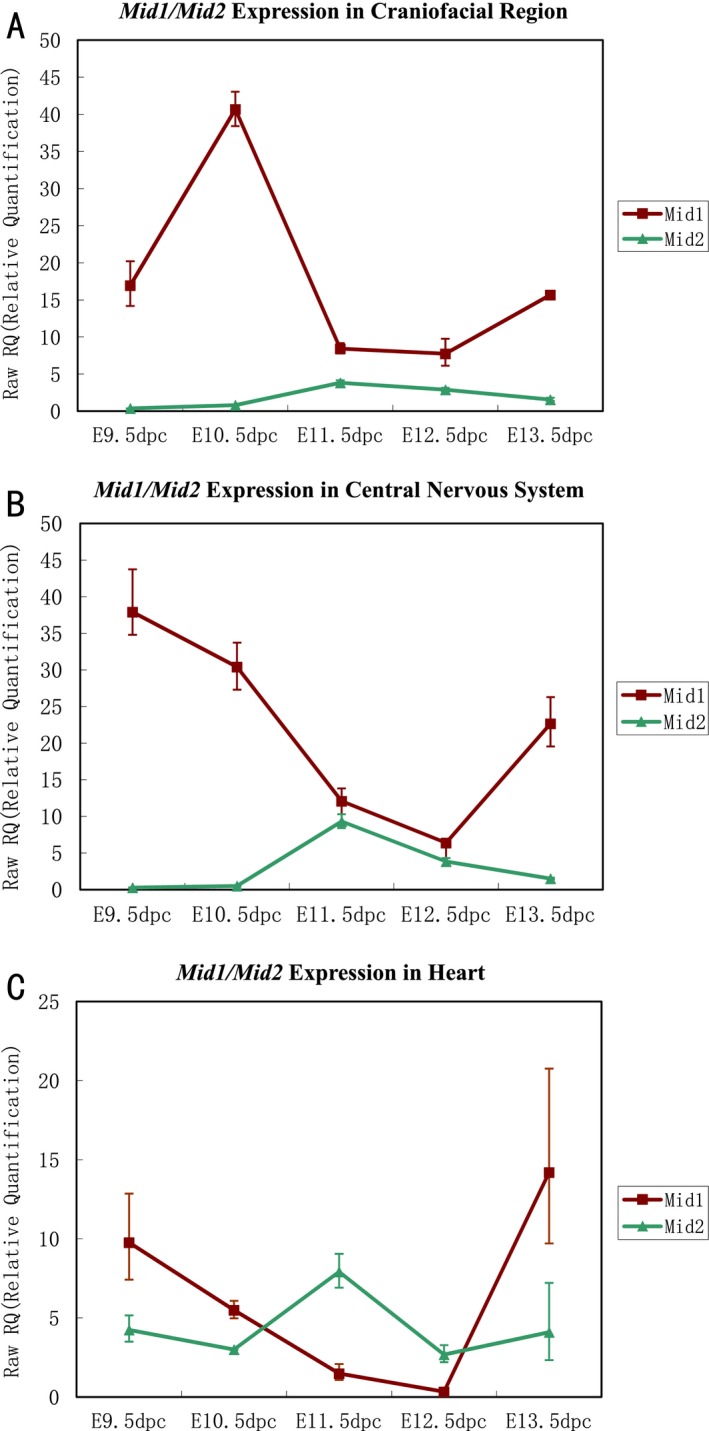
Expressions of *Mid1* and *Mid2* in the major structures of mouse embryos. The expressions of mouse *Mid1* (indicated with red closed squares) and *Mid2* (indicated with closed green triangles) in the craniofacial region, CNS, and heart were determined, using quantitative real‐time RT‐PCR in mouse embryos of different developmental stages. (A) Both *Mid1* and *Mid2* were expressed in the craniofacial regions of mouse embryos from E9.5 dpc to E13.5 dpc. *Mid2* displayed an overall lower level of expression compared with *Mid1*. High expression of *Mid1* was detected at E10.5 dpc and dropped dramatically at E11.5 dpc, while peak expression of *Mid2* appeared at E11.5 dpc. (B) Both *Mid1* and *Mid2* were expressed in the CNS of mouse embryos from E9.5 dpc to E13.5 dpc. *Mid2* displayed an overall lower level of expression compared with *Mid1*. High expression of *Mid1* was detected at E9.5 dpc and dropped dramatically in E11.5 dpc and E12.5 dpc mouse embryos, and significantly increased again at E13.5 dpc. On the contrary, peak expression of *Mid2* was observed in E11.5 dpc mouse embryos. (C) Both *Mid1* and *Mid2* were expressed in mouse embryonic hearts from E9.5 dpc to E 13.5 dpc. Peak expression of *Mid2* appeared at E11.5 dpc, while the expression of *Mid1* dropped to a very low level in E10.5 dpc and E11.5 dpc embryos.

Given the functional similarities and redundancy indicated in previous research, the expression of *Mid2* was determined in the major structures in mouse embryos (from E9.5 dpc to E13.5 dpc) and the relative expression level of *Mid2* was compared with that of *Mid1* by real‐time RT‐PCR. Both *Mid1* and *Mid2* were expressed in embryonic craniofacial structures, CNSs, and heart tubes at different developmental stages tested in this study, although, *Mid2* displayed relatively overall lower expression levels (Fig. 2).

At E10.5 dpc, the highest level of *Mid1* expression was observed in the craniofacial region and downregulated at E11.5 dpc and E12.5 dpc. At E13.5 dpc, strong *Mid1* signals were again observed in the same region (Fig. 2A). On the contrary, *Mid2* appears to be expressed at a relatively low level in the craniofacial region at E10.5 dpc and was upregulated at E11.5 dpc and downregulated at E12.5 dpc and remained at a low level at E13.5 dpc (Fig. 2A). Similar observations were attained in developing CNS and heart tube (Fig. 2B, C). A tendency of a mutually repressive expression pattern between *Mid1* and *Mid2* seemed likely in these structures during early embryogenesis.

**Figure 3:**
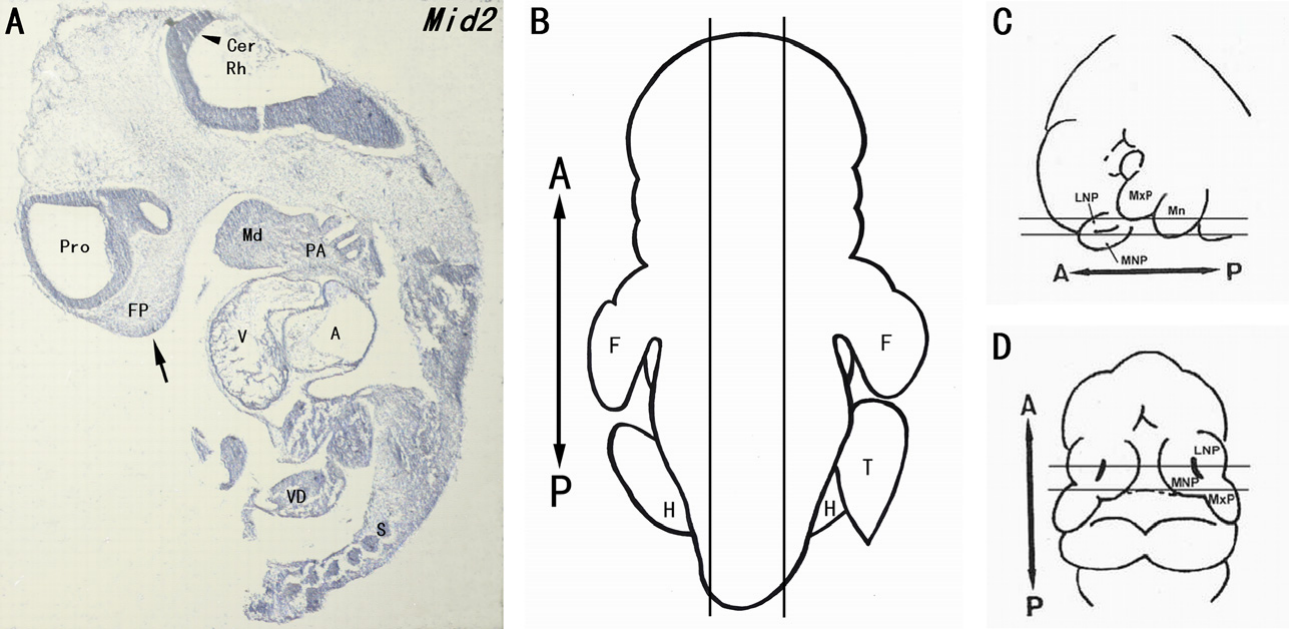
Expression of *Mid2* in E10.5 dpc mouse embryo. (A) RNA in situ hybridization using *Mid2* specific DNA probe was done on the sagittal section of an E10.5 dpc mouse embryo. Mouse *Mid2* was ubiquitously expressed with strong expressions observed in the mandibular process, pharyngeal arches, frontonasal prominence (indicated with arrow), prosencephalon and rhombencephalon (cerebellar plate, indicated with arrowhead), embryonic heart, somites, and vitelline duct. (B, C and D) Schematic figures showing the sagittal section of mouse embryo, frontal and transverse sections of head, respectively (refer to Kosaka et al. 1985). A–P: antero‐posterior plane; A, atrial chamber; Cer, cerebellar plate; F, forelimb; FP, frontonasal prominence; H, hindlimb; Md, mandibular process; PA, pharyngeal arches; Pro, prosencephalon; Rh, rhombencephalon; S, somites; T, tail; V, ventricular chamber; VD, vitelline duct.

A ubiquitous expression of *Mid2* was further confirmed using in situ hybridization on the sagittal section of an E10.5 dpc mouse embryo, in line with the reported expression pattern of *Mid1* in previous study. The expressions of *MID1* have been shown in human, mouse, and chick embryos (Quaderi et al. 1997; Richman et al. 2002; Pinson et al. 2004), and the expression patterns of *MID1* are well conserved across different species. In E10.5 dpc mouse embryo, ubiquitous expression of *Mid1* with a strong expression was detected within the craniofacial primordia, the branchial arches, and the CNS was displayed previously (Quaderi et al. 1997). In this study, we showed that *Mid2* was also ubiquitously expressed in E10.5 dpc mouse embryo, with particularly high expression observed in the epithelium of craniofacial prominences, pharyngeal arches, prosencephalon, rhombencephalon, and the somites (Fig. 3A). Unlike *Mid1*, high expression of *Mid2* was also noticed in the developing heart tube. Moreover, expression of *Mid2* was particularly strong within the anterior portion of the hindbrain that will give rise to the cerebellum at a later stage (Fig. 3A).

### The expressions of *Mid1* and *Mid2* in craniofacial prominences

**Figure 4:**
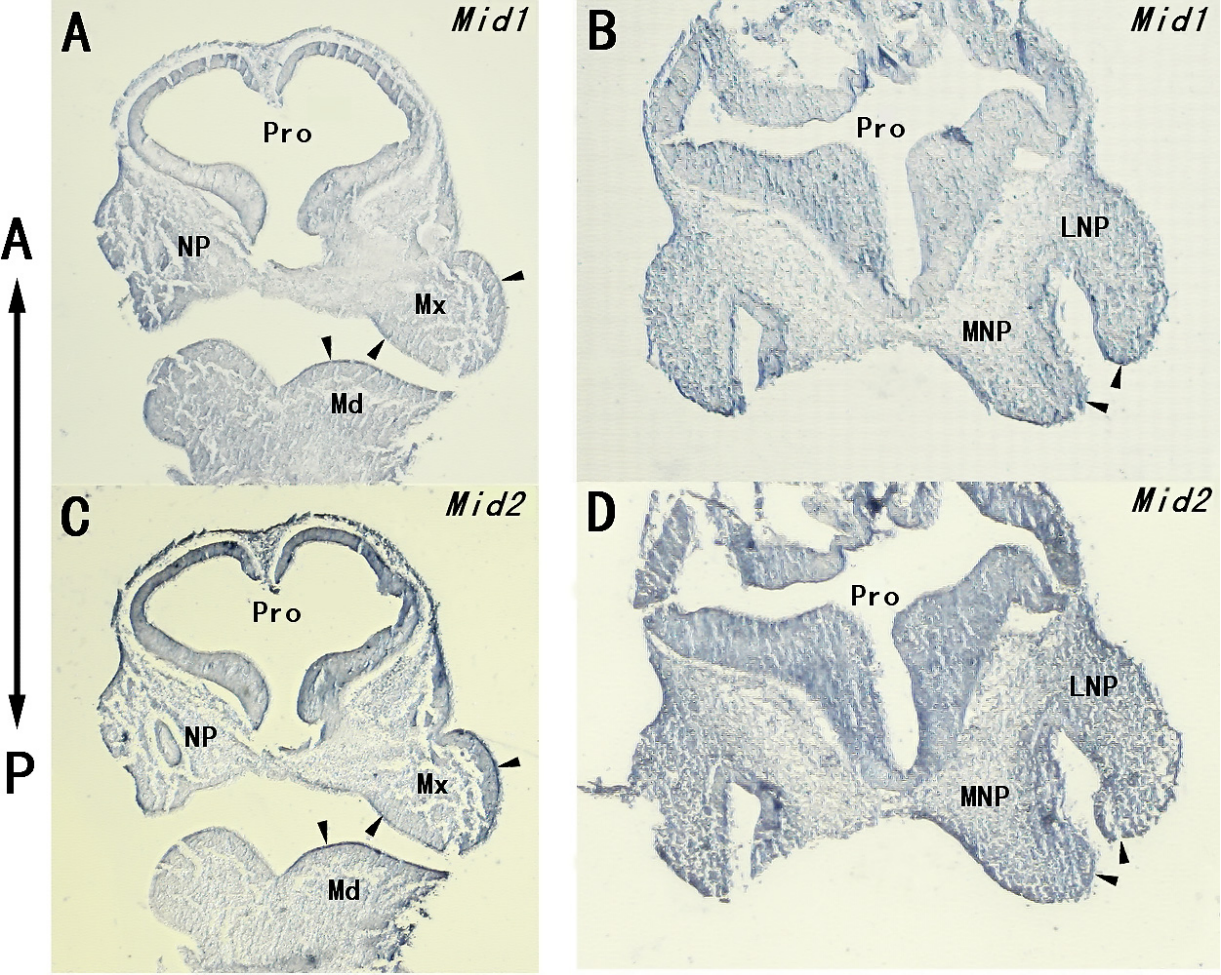
Expressions of *Mid1* and *Mid2* in the craniofacial region of E10.5 dpc mouse embryo. RNA in situ hybridization using *Mid1* and *Mid2‐*specific DNA probes was done on the frontal sections through the head of an E10.5 dpc mouse embryo. Strong expressions of *Mid1* (A) and *Mid2* (C) were observed in the maxillary and mandibular epithelium (arrowheads) and the adjacent mesenchyme. The dorsal‐most border of nasal pit is also evident, and it expressed high levels of *Mid1* and *Mid2*. Strong expressions of *Mid1* (B) and *Mid2* (D) were also detected throughout the LNP and MNP, particularly in the distal ends of these processes (indicated with arrowheads). LNP, lateral nasal process; Md, mandibular process; Mx, maxillary process; MNP, medial nasal process; NP, nasal pit; Pro, prosencephalon.

The detailed expressions of *Mid1* and *Mid2* were further determined in the craniofacial regions of E10.5 dpc mouse embryos. In situ hybridization on the frontal sections through the craniofacial region (as indicated in Fig. 3C) showed strong expressions of *Mid1* (Fig. 4A, B) and *Mid2* (Fig. 4C, D) in the epithelia of the maxillary, mandibular, lateral, and medial nasal processes, as well as in the adjacent mesenchyme of the above‐mentioned structures. The expressions of *Mid1* and *Mid2* were particularly abundant at the distal end of these approaching facial prominences, as indicated with arrowheads. The dorsal‐most border of the nasal pits were also evident on the right side of the frontal section, and high levels of *Mid1/Mid2* expression in these regions were also noticed.

**Figure 5:**
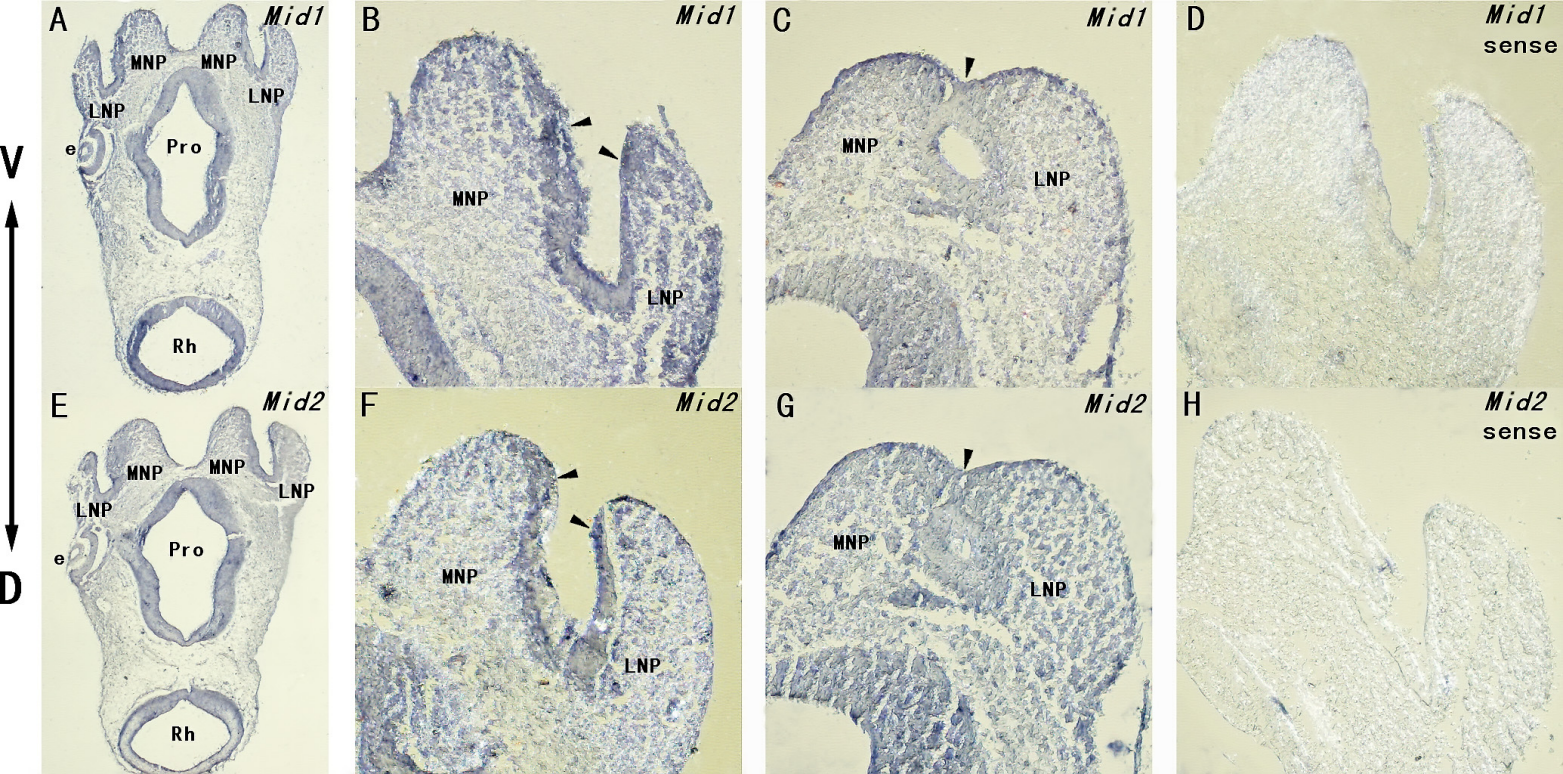
Expressions of *Mid1* and *Mid2* in the craniofacial region of E10.5 dpc mouse embryo before and after fusion.RNA in situ hybridization using *Mid1* and *Mid2* specific DNA probes was done on the transverse sections through the head of an E10.5 dpc mouse embryo. Strong expressions of *Mid1* (A) and *Mid2* (E) were detected in the epithelium and the adjacent mesenchyme of LNP and MNP, particularly in the distal ends of these processes. In magnified view, we observed particularly strong expressions of *Mid1* (B) and *Mid2* (F) at the putative site of fusion (indicated with arrowheads). Expressions of *Mid1* (C) and *Mid2* (G) were dramatically downregulated in the same region after fusion (indicated with arrowheads). No expression was observed using *Mid1* (D) or *Mid2* (H) sense probe as control. e, eye; LNP, lateral nasal process; MNP, medial nasal process; Pro, prosencephalon; Rh, rhombencephalon.

Similar observations were further displayed on the transverse sections through the craniofacial region of E10.5 dpc mouse embryo (as indicated in Fig. 3D). Strong expressions of *Mid1* and *Mid2* were detected in the epithelia and adjacent mesenchyme of the lateral and medial nasal processes, as well as in the epithelia of prosencephalon and rhombencephalon (Fig. 5A, E). In the magnified views, *Mid1/Mid2* expressions were found particularly strong in the epithelia and the underlying mesenchyme at the putative fusion site of approaching lateral and medial nasal processes, while their expressions were significantly downregulated in the epithelial seam of the lateral and medial nasal processes after fusion (Fig. 5B, C, F, G, as indicated with arrowheads).

## Discussion

Opitz syndrome is genetically heterogeneous. The clinical manifestations of OS often consists of hypertelorism/telecanthus, prominent nasal bridge, or depressed nasal root, mild micrognathia, and posteriorly angulated ears with abnormal helices. Structural anomalies of the lip, palate, larynx, trachea, and esophagus are also frequently present. Other defects in OS patients include congenital heart defects, inguinal, umbilical hernias, cryptorchidism, and imperforate/anteriorly displaced anus (McDonald‐McGinn et al. 1995). CNS anomalies, for example, psychomotor delay and mental retardation, have also been reported (Guion‐Almeida and Richieri‐Costa 1992; MacDonald et al. 1993). X‐linked and autosomal dominant OS cannot be distinguished just by clinical manifestations (Robin et al. 1995). Here, in this paper, we reported that hypertelorism remained to be the most common clinical manifestation and was found in all male XLOS patients reviewed, except one. Given the fact that hypertelorism is included in the OS diagnostic criteria, the high incidence of hypertelorism could be an artificial effect due to selective diagnosis. The same bias might also affect the statistics of hypospadias (found in 80% male XLOS), which is also included in the OS diagnostic criteria. The incidence of laryngotracheal defects in our review was lower than that (~60%) reported before since we considered laryngotracheal abnormalities (~47%) and dysphagia/aspiration/gastroesophageal refluxes (~36.4%) as two independent events, with the later being the consequences of both structural defects and malfunctioned nervous system. Brain abnormalities were found in over 50% XLOS reviewed and were slightly higher than that was reported by Fontanella et al. (2008). It could be a consequence of increased MRI examination in XLOS diagnosis in recent years.

The recent review on the *MID1* mutations of XLOS also revealed a lack of genotype–phenotype correlation (Fontanella et al. 2008). In line with the previous findings, the data in this paper revealed no correlation between the types of genetic defects of *MID1* gene and the severities of OS phenotypes. Although mutations in the FNIII domain were suggested to be associate with milder phenotypes, we failed to see this association in our review since three OS cases (OS5 IV‐1, Quaderi et al. 1997; OSP10, Cox et al. 2000; OS308, Migliore et al. 2013) with point mutations in the FNIII domain were noticed suffering with intellectual disabilities along with other severe defects, such as laryngotracheal abnormalities and cardiovascular defects. All *MID1* mutations (point mutations) identified so far occurred on the conserved amino acids between MID1 and MID2 and were scattered throughout the entire length of *MID1* ORF except the RING domain, which was ascribed with E3 ubiquitin‐ligase activity. It indicated the conserved function of MID1 and MID2 and the functional significance of the RING domain, in which mutation might cause fatal death and therefore not found in OS patients. The functional similarities between MID1 and MID2 at the molecular level, including their microtubular distribution, their potential ubiquitin E3 ligase activities, and their functional redundancies in regulating early development, have been demonstrated in previous studies. Further identification of *MID2* gene at the fifth locus for the FG syndrome (Xq22.3), which shares various overlapping clinical features with OS, suggested the clinical significance of *MID2* gene. Recently, a missense mutation in *MID2* gene, p.Arg347Gln, was identified in a large Indian family with X‐linked intellectual disability and it was the first reported disease causative mutation in *MID2*. Minor facial changes in the affected males included short philtrum, prominent ears, and squint, which have all been reported in sporadic XLOS cases (see Table S2). The mild facial changes with MID2 mutation versus the prominent facial abnormalities with MID1 mutations could be partially explained by the relatively lower level of expression of *MID2* in the craniofacial structures. Given the overlapping expression pattern and a tendency of mutually repressed expressions of *Mid1* and *Mid2* in the main embryonic structures including craniofacial structures, CNS, and the heart tube, as revealed by quantitative real‐time PCR in this study, functional redundancy of Mid1 and Mid2 is also supported, as suggested by previous researches.

Considering the commonly seen structural anomalies in OS, such as CLP, heart defects (affected atrioventricular valves and the separation of left/right atrium/ventricles and outflow tracts), and hypospadias, defective outgrowth and fusion events of facial prominences, endocardial cushions, and urethral folds were proposed underlying OS pathogenesis. Further expression studies using in situ hybridization in this research showed that *Mid2* was expressed in the epithelium and in the adjacent mesenchyme of craniofacial prominence in E10.5 dpc mouse embryos, in line with the expression pattern of *Mid1*. Downregulation of *Mid1* and *Mid2* expression in the facial epithelial seam after fusion indicated likely roles of Mid1 and Mid2 in regulating epithelial‐mesenchymal transformation (EMT), a fundamental process in embryonic fusion. This finding was in line with our unpublished data showing that MID2 might act as a regulatory factor that inhibited EMT since treatment of *MID2* antisense oligo significantly increased the activation of embryonic cardiac endothelial cells in chick embryos and their mesenchymal invasion. Since several signalling pathways, including TGF‐beta/BMP, Wnt/beta‐catenin, and Notch, have all been implicated in regulating EMT (Armstrong and Bischoff 2004), cross‐talks between MID1/MID2 and these signalling pathways await future investigations.

## Conflict of Interest

None declared.

## Supporting information


**Table S1.** Clinical findings of OS patients with *MID1* gene mutations.

**Table S2.** Craniofacial features and oral cavity abnormalities in patients with *MID1* gene mutations.

**Table S3.** Central nervous system defects in patients with *MID1* gene mutations.

**Table S4.** Heart defects in patients with *MID1* gene mutations.

**Table S5.** Clinical findings in patients with *MID2* gene mutation.
