# Supplementary material for: Mid1/Mid2 expression in craniofacial development and a literature review of X‐linked opitz syndrome
Source: Mol Genet Genomic Med. 2015 Dec 12;4(1):95–105. doi: 10.1002/mgg3.183 (PMC4707030; doi:10.1002/mgg3.183)
Supplement: Supplementary file 1 — Table S1. Clinical findings of OS patients with MID1 gene mutations. [file MGG3-4-095-s001.doc]

**Supp. Table S1. Clinical Findings of OS patients with *MID1* Gene Mutations**

| Literature cases | | Sex | Genetic finding | Impact on protein  structure | Craniofacial abnormalities | | | | Laryngotracheal abnormalities | Dysphagia/ aspiration/ gastroesophageal reflux | Intellectual disability/  MR/ Dev  delay | Brain abnormalities | Cardiovascular defects | Urogenital defects | |
| --- | --- | --- | --- | --- | --- | --- | --- | --- | --- | --- | --- | --- | --- | --- | --- |
|  |  |  |  |  | Hypertelorism | Anteverted nares | CL/P | Ear anomalies |  |  |  |  |  | Hypospadias | Anal defects |
| Quaderi et al. [1997]  Robin et al. [1995] | OS5 Ⅳ-1 | M | c.1312_1314delATG | p.Met438del | + | + | + |  | + |  | + | + |  | + | + |
| Quaderi et al. [1997]  Robin et al. [1995] | OS5 Ⅳ-2 | F |  |  | + |  |  |  |  |  | + |  |  |  | + |
| Quaderi et al. [1997]  Robin et al. [1995] | OS5 Ⅲ-2 | F |  |  | + |  |  |  |  |  |  |  |  |  |  |
| Quaderi et al. [1997]  Robin et al. [1995] | OS5 Ⅲ-4 | F |  |  | + |  |  |  |  | + |  |  |  |  |  |
| Quaderi et al. [1997] | OS16 two  brothers | M | c.1558dupG | p.Glu520GlyfsX18 | + |  | + |  |  |  | Mild |  |  | + |  |
|  |  | M |  |  | + |  | + |  |  |  | Mild |  |  | + |  |
| Quaderi et al. [1997] | OS16 mother | F |  |  | + |  |  |  |  |  | - |  |  |  |  |
| Quaderi et al. [1997] | OS20 | M | c.1601_1624dup24 | p.Tyr542LeuinsFIDSGRHY | + |  |  |  |  | + | Mild |  |  | + |  |
| Quaderi et al. [1997] | OS20 mother | F |  |  | + |  |  |  |  |  |  |  |  |  |  |
| Quaderi et al. [1997] | OS20 half  sister | F |  |  | + |  |  |  |  |  |  |  |  |  |  |
| Gaudenz et al. [1998]  Brooks et al. [1992] | OS3 | M | c.796T>C | p.Cys266Arg | + | + | + | + | + | + | - |  | - | + |  |
| Gaudenz et al. [1998]  Brooks et al. [1992] | OS3 mother | F |  |  | + | + |  |  |  |  |  |  |  |  |  |
| Gaudenz et al. [1998] | OS14 | M | c.949delG | p.Glu317AsnfsX4 | + |  |  |  |  |  |  |  |  | + | + |
| Gaudenz et al. [1998] | OS14 mother | F |  |  |  |  |  |  |  |  |  |  |  |  |  |
| Gaudenz et al. [1998] | OS19 two  brothers | M | c.1286-2A>G | r.(spl?) | + |  |  |  |  |  |  |  |  | + | + |
|  |  | M |  |  | + |  | + |  |  |  |  |  |  | + |  |
| Gaudenz et al. [1998] | OS19 mother | F |  |  | + |  |  |  |  |  |  |  |  |  |  |
| Gaudenz et al. [1998] | OS19 sister | F |  |  | + |  |  |  |  |  |  |  |  |  |  |
| Gaudenz et al. [1998] | OS28 | M | c.1483ins160 | p.Arg495?fsX6 | + |  | + |  | + |  |  |  |  |  |  |
| Gaudenz et al. [1998] | OS28 two  females | F |  |  | + |  |  |  |  |  |  |  |  |  |  |
|  |  | F |  |  | + |  |  |  |  |  |  |  |  |  |  |
| Gaudenz et al. [1998] | OS36 | M | c.1607T>C | p.Ile536Thr | + |  | + |  | + | + |  |  | + | + |  |
| Gaudenz et al. [1998] | OS36 mother | F |  |  | + |  |  |  |  |  |  |  |  |  |  |

**Supp. Table S1. Continued**

| Literature cases | | Sex | Genetic finding | Impact on protein  structure | Craniofacial abnormalities | | | | Laryngotracheal abnormalities | Dysphagia/ aspiration/ gastroesophageal reflux | Intellectual disability/  MR/ Dev  delay | Brain abnormalities | Cardiovascular defects | Urogenital defects | |
| --- | --- | --- | --- | --- | --- | --- | --- | --- | --- | --- | --- | --- | --- | --- | --- |
|  |  |  |  |  | Hypertelorism | Anteverted nares | CL/P | Ear anomalies |  |  |  |  |  | Hypospadias | Anal defects |
| Gaudenz et al. [1998] | Patient with sporadic OS | M | c.1527ins13bp | p.Glu507SerfsX4 | + |  |  |  |  |  |  |  |  | + |  |
| Schweiger et at. [1999] | Proband SW | M | c.1800_1803delCCTC | p.Leu601GlyfsX20 |  |  |  |  |  |  |  |  |  |  |  |
| Schweiger et at. [1999] | Mother | F |  |  |  |  |  |  |  |  |  |  |  |  |  |
| Cox et al. [2000] | OSP3 | M | c.1102C>T | p.Arg368X | + | + | + | + | - | + | - | - | - | + | - |
| Cox et al. [2000] | OSP5 | M | c.1877T>C | p.Leu626Pro | + | + | + | + | - | - | - | - | + | + | - |
| Cox et al. [2000] | OSP6 | M | c.1052delC | p.Pro351LeufsX14 | + |  | + | + | - | + | + | - | + | + | - |
| Cox et al. [2000] | OSP9 | M | c.1483C>T | p.Arg495X | + | + | - | + | + | + | + | + | + | + | + |
| Cox et al. [2000] | OSP10 | M | c.1402C>T | p.Gln468X | + | + | - | + | + | + | + | + | + | - | - |
| Cox et al. [2000] | OSP11 | M | c.661-?_756+?del | p.Gln221_Glu252del | + | + | - | + | + | + | + | - | - | + | - |
| Cox et al. [2000] | OSP12 | M | c.343G>T | p.Glu115X | + |  | + | + | + | + | + | - | - | - | + |
| Winter et al. [2003] | Family 1  proband | M | c.1655+1G>A | r.(spl?) | + |  | + |  |  |  |  |  |  |  |  |
| Winter et al. [2003] | Family 1 two  maternal cousins | M |  |  | + |  |  |  |  |  |  |  |  | + |  |
|  |  | M |  |  | + |  |  |  |  |  |  |  |  | + |  |
| Winter et al. [2003] | Family 1  maternal uncle | M |  |  |  |  | + |  |  |  |  |  |  |  |  |
| Winter et al. [2003] | Family 2  brother | M | c.1448-1A>G | r.(spl?) |  |  |  |  |  |  |  |  |  |  |  |
| Winter et al. [2003] | Family 2  sister | F |  |  |  |  |  |  |  |  |  |  |  |  |  |
| Winter et al. [2003] | Family 3  proband | M | c.1-?_ 660+?dup | r.(spl?)/p.? | + |  |  | + | + |  |  |  | + |  | + |
| Winter et al. [2003] | Family 3  mother | F |  |  | + |  |  |  | + |  |  |  |  |  |  |
| De Falco et al. [2003] | O2 | M | c.1387G>T | p.Val463Phe | + | + | + | - | + | + | Mild |  | - | + | - |
| De Falco et al. [2003] | O2 mother | F |  |  | + |  |  |  |  |  |  |  |  |  |  |
| De Falco et al. [2003] | O5 | M | c.1331dupA | p.Gln445AlafsX31 | + |  | + |  | + |  | - |  | - | + | - |
| De Falco et al. [2003] | O9 | M | c.1483C>T | p.Arg495X | + | + | + | + | + |  |  | + | - | + | + |
| De Falco et al. [2003] | O10 | M | c.1483C>T | p.Arg495X | + | + | - | - | + |  | - | - | - | + | - |
| De Falco et al. [2003] | O10 maternal uncle | M |  |  | + |  | + |  |  |  |  |  |  | + |  |

**Supp. Table S1. Continued**

| Literature cases | | Sex | Genetic finding | Impact on protein  structure | Craniofacial abnormalities | | | | Laryngotracheal abnormalities | Dysphagia/ aspiration/ gastroesophageal reflux | Intellectual disability/  MR/ Dev  delay | Brain abnormalities | Cardiovascular defects | Urogenital defects | |
| --- | --- | --- | --- | --- | --- | --- | --- | --- | --- | --- | --- | --- | --- | --- | --- |
|  |  |  |  |  | Hypertelorism | Anteverted nares | CL/P | Ear anomalies |  |  |  |  |  | Hypospadias | Anal defects |
| De Falco et al. [2003] | O10 mother | F |  |  | + |  |  |  |  |  |  |  |  |  |  |
| De Falco et al. [2003] | O10 maternal grandmother | F |  |  |  |  |  |  |  |  |  |  |  |  |  |
| De Falco et al. [2003] | O16 twins | M | c.757-1G>C | r.(spl?) | + | + | + |  | + | + | Mild |  | + | + | - |
|  |  | M |  |  | + | + | + |  | + | + | Mild | + | + | + | - |
| De Falco et al. [2003] | O16 brother | M |  |  | + |  | + |  | + |  |  |  | + | + | - |
| De Falco et al. [2003] | O16 mother | F |  |  | + |  |  |  |  |  |  |  |  |  |  |
| De Falco et al. [2003] | O22 | M | c.1286-1G>A | r.(spl?) | + |  |  |  |  |  | Mild |  | + | + |  |
| De Falco et al. [2003] | O26 | M | c.1547_1551dupGTCAC | p.Thr518ValfsX15 | + | - | - | - | + | + | Mild |  | - | + | + |
| De Falco et al. [2003] | O26 mother | F |  |  | + |  |  |  |  |  |  |  |  |  |  |
| De Falco et al. [2003] | O34 | M | c.584G>T | p.Cys195Phe | + | - | - | - |  | + | + | - | - | + | + |
| De Falco et al. [2003] | O34 mother | F |  |  | + |  |  |  |  |  |  |  |  |  |  |
| De Falco et al. [2003] | O36 | M | c.1039C>T | p.Gln347X | + | - | - | - | + |  | - | + | + | + | + |
| De Falco et al. [2003] | O36 brother | M |  |  | + |  |  |  | + |  |  | + |  |  |  |
| De Falco et al. [2003] | O36 mother | F |  |  | + |  |  |  |  |  |  |  |  |  |  |
| De Falco et al. [2003] | O42 | M | c.1106_1107delAG | p.Lys370GlufsX18 | + |  | + | + | + |  | + |  | + | + |  |
| De Falco et al. [2003] | O43 | M | c.1267G>T | p.Gly423X | + |  | - | + | + |  | - |  | - | - |  |
| Pinson et al. [2004] | Family 1  proband | M | c.1354G>A | p.Gly452Ser | + | - | - | + | + | + | - | - | - | + | - |
| Pinson et al. [2004] | Family 1  brother | M |  |  | + | - | - | + |  | + | - | - | - | - | - |
| Pinson et al. [2004] | Family 1  mother | F |  |  | + |  |  |  |  |  |  |  |  |  |  |
| Pinson et al. [2004] | Family 2  proband | M | c.829C>T | p.Arg277X | + | + | + | + | + |  | + | + | - | + | - |
| Pinson et al. [2004] | Family 2  maternal half-brother | M |  |  | + | - | + | + | + |  | + |  | - | + | - |
| Pinson et al. [2004] | Family 2  mother | F |  |  | - | - | - | - | - | - | - | - | - | - | - |

**Supp. Table S1. Continued**

| Literature cases | | Sex | Genetic finding | Impact on protein  structure | Craniofacial abnormalities | | | | Laryngotracheal abnormalities | Dysphagia/ aspiration/ gastroesophageal reflux | Intellectual disability/  MR/ Dev  delay | Brain abnormalities | Cardiovascular defects | Urogenital defects | |
| --- | --- | --- | --- | --- | --- | --- | --- | --- | --- | --- | --- | --- | --- | --- | --- |
|  |  |  |  |  | Hypertelorism | Anteverted nares | CL/P | Ear anomalies |  |  |  |  |  | Hypospadias | Anal defects |
| Pinson et al. [2004] | Patient 3 | M | c.1285+3_+6delGAGT | r.(spl?) | + | + | - | - | + | + | Mild | - | + | + | - |
| Pinson et al. [2004] | Patient 3  mother | F |  |  | + |  |  |  |  |  |  |  |  |  |  |
| Pinson et al. [2004] | Patient 4 | M | c.1444_1447dupAACA | p.Ser483LysfsX6 | + | + | - | + | + | + | - | + | - | + | + |
| Pinson et al. [2004] | Family 6  proband | M | c.1483C>T | p.Arg495X | + | + | + | + |  | + | + | + | - | + | + |
| Pinson et al. [2004] | Family 6  brother | M |  |  | + | + | - |  |  | + | + | + | + | + | - |
| Pinson et al. [2004] | Family 6  mother | F |  |  |  |  |  |  |  |  |  |  |  |  |  |
| Pinson et al. [2004] | Patient 9 | M | c.403_411del9 | p.Val135_Cys137del | + | - | - | + |  | + | - |  | - | + | - |
| So et al. [2005] | OSF4 | M | c.884T>C | p.Leu295Pro | + |  | + |  |  |  |  |  |  | + |  |
| So et al. [2005] | OSF4 brother | M |  |  | + |  |  |  |  |  |  |  |  | + |  |
| So et al. [2005] | OSF4 mother | F |  |  | + |  |  |  |  |  |  |  |  |  |  |
| So et al. [2005] | OSF6 | M | c.1545_1546delGA | p.Pro519X | + |  | + |  | + |  |  |  | + | + |  |
| So et al. [2005] | OSF6 maternal grandfather | M |  |  | + |  |  |  | + | + |  |  |  |  | + |
| So et al. [2005] | OSF6 maternal cousin | M |  |  |  |  |  |  |  |  |  |  |  |  |  |
| So et al. [2005] | OSF6 mother | F |  |  | + |  |  |  |  |  |  |  |  |  |  |
| So et al. [2005] | OSF7 | M | c.1172_1174delTCT | p.Leu391_Cys392delinsArg | + |  |  |  |  |  |  |  |  | + | + |
| So et al. [2005] | OSF7 mother | F |  |  | + |  |  |  |  |  |  |  |  |  |  |
| So et al. [2005] | OSF11 | M | c.1656G>A | p.Trp552X | + |  | + |  |  |  |  |  |  | + | + |
| So et al. [2005] | OSF11 mother | F |  |  | + |  |  |  |  |  |  |  |  |  |  |
| So et al. [2005] | OSF23 | M | c.865-1G>T | r.(spl?) | + |  | + | + |  |  | + |  |  | + |  |
| So et al. [2005] | OSF23 mother | F |  |  | + |  |  |  |  |  |  |  |  |  |  |
| So et al. [2005] | Patient 14 | M | c.1447+1G>A | r.(spl?) | + |  | + |  |  |  |  |  |  |  |  |
| So et al. [2005] | Patient 25 | M | c.1442_1445dupCAAA | p.Ser483LysfsX6 | + |  |  | + |  | + | + |  |  | + |  |

**Supp. Table S1. Continued**

| Literature cases | | Sex | Genetic finding | Impact on protein  structure | Craniofacial abnormalities | | | | Laryngotracheal abnormalities | Dysphagia/ aspiration/ gastroesophageal reflux | Intellectual disability/  MR/ Dev  delay | Brain abnormalities | Cardiovascular defects | Urogenital defects | |
| --- | --- | --- | --- | --- | --- | --- | --- | --- | --- | --- | --- | --- | --- | --- | --- |
|  |  |  |  |  | Hypertelorism | Anteverted nares | CL/P | Ear anomalies |  |  |  |  |  | Hypospadias | Anal defects |
| So et al. [2005] | Patient 46 | M | c.1313_1316delTGAT | p.Met438LysfsX41 | + |  |  | + |  |  | Mild |  |  | + |  |
| So et al. [2005] | Patient 62 | M | c.1448-8_1459insdel20 | r.(spl?) | + |  | + | + |  |  |  |  |  | + |  |
| So et al. [2005] | Patient 75 | M | c.829C>T | p.Arg277X | + |  | + |  |  | + |  |  |  | + |  |
| So et al. [2005] | Patient 75  maternal cousin | M |  |  | + |  |  |  |  |  |  |  |  | + |  |
| So et al. [2005] | Patient 75  maternal cousin | M |  |  | + |  |  |  |  | + |  |  |  |  |  |
| Shaw et al. [2006] | Proband | M | c.1483C>T | p.Arg495X | + |  | + |  |  |  | Mild | - | + | + |  |
| Shaw et al. [2006] | Brother | M |  |  | + |  |  |  |  |  |  |  |  | + |  |
| Shaw et al. [2006] | Mother | F |  |  | + |  |  |  |  |  |  |  |  |  |  |
| Shaw et al. [2006] | Maternal grandmother | F |  |  | + |  |  |  |  |  |  |  |  |  |  |
| Mnayer et al. [2006] | Patient | M | c.1322C>T | p.Pro441Leu | + |  | - | + | + |  | - | - | - | + | - |
| Cho et al. [2006] | Proband | M | c.1798dupC | p.His600ProfsX12 | + | - | - | - | + | + | - | - | - | + | - |
| Cho et al. [2006] | Brother | M |  |  |  |  |  |  |  |  |  |  |  |  |  |
| Cho et al. [2006] | Sister | F |  |  |  |  |  |  |  |  |  |  |  |  |  |
| Cho et al. [2006] | Mother | F |  |  | + |  |  |  |  |  |  |  |  |  |  |
| Ferrentino et al. [2007] Fontanella et al. [2008] | OS8 | M | c.434G>A | p.Cys145Tyr |  |  | + |  |  |  |  |  |  | + |  |
| Ferrentino et al. [2007] Fontanella et al. [2008] | OS8 maternal half-brother | M |  |  |  |  |  |  |  |  |  |  |  | + | + |
| Ferrentino et al. [2007] | OS89 | M | c.1798delC | p.His600ThrfsX22 |  |  |  |  |  |  |  |  |  |  |  |
| Ferrentino et al. [2007] Fontanella et al. [2008] | OS98 | M | c.1-?_2004+?del | p.? | + |  | - |  |  |  | Mild | - |  | + |  |
| Ferrentino et al. [2007] Fontanella et al. [2008] | OS98 brother | M |  |  | + |  | - |  |  |  | - |  |  | + |  |
| Ferrentino et al. [2007] Fontanella et al. [2008] | OS112 | M | c.1285+1G>T | r.(spl?) | + |  | + |  |  |  |  |  | - | + |  |
| Ferrentino et al. [2007] Fontanella et al. [2008] | OS112 mother | F |  |  | + |  |  |  |  |  |  |  |  |  |  |

**Supp. Table S1. Continued**

| Literature cases | | Sex | Genetic finding | Impact on protein  structure | Craniofacial abnormalities | | | | Laryngotracheal abnormalities | Dysphagia/ aspiration/ gastroesophageal reflux | Intellectual disability/  MR/ Dev  delay | Brain abnormalities | Cardiovascular defects | Urogenital defects | |
| --- | --- | --- | --- | --- | --- | --- | --- | --- | --- | --- | --- | --- | --- | --- | --- |
|  |  |  |  |  | Hypertelorism | Anteverted nares | CL/P | Ear anomalies |  |  |  |  |  | Hypospadias | Anal defects |
| Ferrentino et al. [2007] Fontanella et al. [2008] | OS118 | M | c.397_401delACCTG | p.Thr133CysfsX4 | + |  |  |  |  |  |  |  |  | + |  |
| Ferrentino et al. [2007] Fontanella et al. [2008] | OS118 mother | F |  |  | + |  |  |  |  |  |  |  |  |  |  |
| Ferrentino et al. [2007] Fontanella et al. [2008] | OS136 | M | c.1832A>G | p.Asp611Gly | + |  |  |  | +* | +* |  |  |  |  |  |
| Ferrentino et al. [2007] Fontanella et al. [2008] | OS136maternal grandfather | M |  |  | + |  |  |  |  |  |  |  |  |  |  |
| Ferrentino et al. [2007] Fontanella et al. [2008] | OS136maternal uncle | M |  |  |  |  |  |  | +* | +* |  |  |  |  | + |
| Ferrentino et al. [2007] Fontanella et al. [2008] | OS136maternal cousin | M |  |  |  |  | + |  |  |  |  |  |  |  |  |
| Ferrentino et al. [2007] Fontanella et al. [2008] | OS149 | M | c.819_829del11 | p.Ile273MetfsX31 | + |  |  |  |  |  | - | + |  | + | + |
| Ferrentino et al. [2007] | OS157 | M | c.864+1G>T | r.(spl?) |  |  |  |  |  |  |  |  |  |  |  |
| Ferrentino et al. [2007] | OS157 mother | F |  |  | + |  |  |  |  |  |  |  |  |  |  |
| Ferrentino et al. [2007] Fontanella et al. [2008] | OS159 | M | c.388G>A | p.Ala130Thr | - |  |  |  | +* | +* |  | - | - | + |  |
| Ferrentino et al. [2007] | OS165 | M | c.1141+2T>C | r.(spl?) |  |  |  |  |  |  |  |  |  |  |  |
| Ferrentino et al. [2007] | OS165 mother | F |  |  | + |  |  |  |  |  |  |  |  |  |  |
| Ferrentino et al. [2007] Fontanella et al. [2008] | OS167 | M | c.1573C>T | p.Gln525X | + |  | + |  | +* | +* |  |  |  | + |  |
| Ferrentino et al. [2007] Fontanella et al. [2008] | OS167 mother | F |  |  | + |  |  |  |  |  |  |  |  |  |  |
| Ferrentino et al. [2007] Fontanella et al. [2008] | OS168 | M | c.1-?_ 660+?del | p.? | + |  |  |  | +* | +* | - | - | + | + |  |
| Ferrentino et al. [2007] Fontanella et al. [2008] | OS168 maternal uncle | M |  |  | + |  |  |  |  |  |  |  | + | + |  |
| Ferrentino et al. [2007] Fontanella et al. [2008] | OS172 | M | c.712G>T | p.Glu238X | + |  |  |  | +* | +* |  |  |  | + | + |
| Ferrentino et al. [2007] Fontanella et al. [2008] | OS172 maternal uncle | M |  |  | + |  |  |  |  |  |  |  |  | + |  |

**Supp. Table S1. Continued**

| Literature cases | | Sex | Genetic finding | Impact on protein  structure | Craniofacial abnormalities | | | | Laryngotracheal abnormalities | Dysphagia/ aspiration/ gastroesophageal reflux | Intellectual disability/  MR/ Dev  delay | Brain abnormalities | Cardiovascular defects | Urogenital defects | |
| --- | --- | --- | --- | --- | --- | --- | --- | --- | --- | --- | --- | --- | --- | --- | --- |
|  |  |  |  |  | Hypertelorism | Anteverted nares | CL/P | Ear anomalies |  |  |  |  |  | Hypospadias | Anal defects |
| Ferrentino et al. [2007] Fontanella et al. [2008] | OS173 | M | c.1856_1858delATG | p.Asp619del | + |  | + |  |  |  |  | - | + | + |  |
| Ferrentino et al. [2007] Fontanella et al. [2008] | OS174 | M | c.1957G>A | p.Gly653Arg | + |  | + |  |  |  |  |  |  | + |  |
| Ferrentino et al. [2007] | OS179 | M | c.1608_1611dupTGAT | p.Ser538X |  |  |  |  |  |  |  |  |  |  |  |
| Ferrentino et al. [2007] Fontanella et al. [2008] |  | M | c.1-?_2004+?del | p.? |  |  | + |  | +* | +* |  |  |  | + |  |
| Ferrentino et al. [2007] Fontanella et al. [2008] | OS195 | M | c.951dupA | p.His318ThrfsX7 | + |  | + |  | +* | +* |  |  |  |  |  |
| Ferrentino et al. [2007] Fontanella et al. [2008] |  | M | c.1-?_2004+?del | p.? | + |  | + |  | +* | +* |  |  |  | + |  |
| Ferrentino et al. [2007] Fontanella et al. [2008] | OS216 | M | c.1108A>G | p.Lys370Glu |  |  | + |  |  |  |  |  |  | + |  |
| Ferrentino et al. [2007] Fontanella et al. [2008] | OS220 | M | c.389C>T | p.Ala130Val | + |  | + |  | +* | +* |  |  |  |  |  |
| Ferrentino et al. [2007] Fontanella et al. [2008] | OS223 | M | c.561T>A | p.Cys187X | + |  | + |  |  |  |  |  |  | + |  |
| Ferrentino et al. [2007] Fontanella et al. [2008] | OS227 | M | c.1602_1606dupGTTT | p.Ile536ValfsX3 | + |  |  |  |  |  |  |  |  | + |  |
| Ferrentino et al. [2007] Fontanella et al. [2008] | OS229 | M | c.1594G>A | p.Gly532Arg | + |  |  |  | - |  | + |  | - | - |  |
| Ferrentino et al. [2007] Fontanella et al. [2008] | OS229 brother | M |  |  | + |  |  |  | +* | +* | Mild |  | + | - |  |
| Ferrentino et al. [2007] Fontanella et al. [2008] | OS231 | M | c.1452_1455delACCA | p.Gln484HisfsX13 | + |  | + |  | +* | +* |  | + | + | + |  |
| Ferrentino et al. [2007] Fontanella et al. [2008] | OS231 mother | F |  |  | + |  |  |  |  |  |  |  |  |  |  |
| Ferrentino et al. [2007] Fontanella et al. [2008] | OS232 | M | c.430G>T | p.Glu144X | + |  | - |  | +* | +* |  |  | - | + | - |
| Ferrentino et al. [2007] Fontanella et al. [2008] | OS235 | M | c.425G>C | p.Cys142Ser |  |  | + |  |  |  |  |  |  | + |  |

**Supp. Table S1. Continued**

| Literature cases | | Sex | Genetic finding | Impact on protein  structure | Craniofacial abnormalities | | | | Laryngotracheal abnormalities | Dysphagia/ aspiration/ gastroesophageal reflux | Intellectual disability/  MR/ Dev  delay | Brain abnormalities | Cardiovascular defects | Urogenital defects | |
| --- | --- | --- | --- | --- | --- | --- | --- | --- | --- | --- | --- | --- | --- | --- | --- |
|  |  |  |  |  | Hypertelorism | Anteverted nares | CL/P | Ear anomalies |  |  |  |  |  | Hypospadias | Anal defects |
| Fontanella et al. [2008] | OS235maternal nephew | M |  |  | + |  |  |  | +* | +* |  |  | + | + |  |
| Ferrentino et al. [2007] | OS247 | M | c.1491_1533dup43 | p.Ser512GlufsX5 |  |  |  |  |  |  |  |  |  |  |  |
| Ferrentino et al. [2007] | OS248 | M | c.1663A>G | p.Ile555Val |  |  |  |  |  |  |  |  |  |  |  |
| Fontanella et al. [2008] |  | M | c.829C>T | p.Arg277X |  |  |  |  |  |  |  |  |  |  |  |
| Fontanella et al. [2008] | Proband | M | c.606delG | p.Arg203GlyfsX10 | + |  |  |  | +* | +* | + |  |  | + |  |
| Fontanella et al. [2008] | Brother | M |  |  | + |  |  |  | +* | +* | - |  | + | + |  |
| Fontanella et al. [2008] | Patient with sporadic OS | M | c.452C>T | p.Pro151Leu | + |  |  |  |  |  | + |  |  | + |  |
| Hsieh et al. [2008] | Patient 6 | M | c.1540ins13bp | p.Lys514X | + |  | + | + |  | + | + |  | - | + |  |
| Hu et al. [2012] | Patient | M | c.1703T>C | p.Ile568Thr | + |  | + |  |  |  |  | + | - | + |  |
| Huning et al. [2013] | Patient | M | arr snp  Xp22.2(10,466,103-10,523,151)x2 mat  (NCBI37/hg19) | p.Gln221_Glu252dup | + |  |  | + | - | + | - | + | - | - |  |
| Huning et al. [2013] | Mother | F |  |  | + |  |  |  |  | + |  |  |  |  |  |
| Migliore et al. [2013] | OS226 | M | c.-56-?_660+?del | p.? | + |  | - | + | - |  | - | + | - | + |  |
| Migliore et al. [2013] | OS242 | M | c.-400_660+? (var1) c.-330_660+? (var4) | p.? | + |  | + | + | + | + | - | - | - | - | - |
| Migliore et al. [2013] | OS280 | M | c.865-?_1013+?del | p.Val289SerfsX14 | + |  | - |  | + | + | - |  | - | - |  |
| Migliore et al. [2013] | OS280 brother | M |  |  |  |  |  |  |  |  |  |  |  |  | + |
| Migliore et al. [2013] | OS280 maternal cousin | M |  |  |  |  |  |  | + |  |  |  |  |  |  |
| Migliore et al. [2013] | OS283 | M | c.446C>T | p.Thr149Ile | + |  | + | + | - | + | Mild | - | - | + | - |
| Migliore et al. [2013] | OS291 | M | c.958_959delCT | p.Leu320GlufsX4 | + |  | + | - | - | + | - |  | - | + | - |
| Migliore et al. [2013] | OS291 mother | F |  |  |  |  |  |  |  |  |  |  |  |  |  |
| Migliore et al. [2013] | OS300 | M | c.1176C>A | p.Cys392X | + |  | - | - | + |  |  | + | + | + |  |
| Migliore et al. [2013] | OS308 | M | c.1376_1377insA | p.Tyr459X | + |  | - | + | + |  | + | + | - | + |  |
| Migliore et al. [2013] | OS308 mother | F |  |  |  |  |  |  |  |  |  |  |  |  |  |
| Migliore et al. [2013] | OS313 | M | c.368dupA | p.Asp123GlufsX16 | + |  | + | + | + |  |  | - | - | + | + |
| Migliore et al. [2013] | OS313 brother | M |  |  |  |  | + |  | - |  |  | - | - | + |  |

**Supp. Table S1. Continued**

| Literature cases | | Sex | Genetic finding | Impact on protein  structure | Craniofacial abnormalities | | | | Laryngotracheal abnormalities | Dysphagia/ aspiration/ gastroesophageal reflux | Intellectual disability/  MR/ Dev  delay | Brain abnormalities | Cardiovascular defects | Urogenital defects | |
| --- | --- | --- | --- | --- | --- | --- | --- | --- | --- | --- | --- | --- | --- | --- | --- |
|  |  |  |  |  | Hypertelorism | Anteverted nares | CL/P | Ear anomalies |  |  |  |  |  | Hypospadias | Anal defects |
| Migliore et al. [2013] | OS314 | M | c.1850T>C | p.Phe617Ser | + |  | + | + | - |  | - | + | + | + | - |
| Migliore et al. [2013] | OS314 mother | F |  |  |  |  |  |  |  |  |  |  |  |  |  |
| Migliore et al. [2013] | OS319 | M | c.1323delC | p.Asn442ThrfsX38 | + |  | - |  | + |  | - |  | + | + | + |
| Migliore et al. [2013] | OS319 mother | F |  |  |  |  |  |  |  |  |  |  |  |  |  |
| Migliore et al. [2013] | OS323 | M | c.1444_1447dupAACA | p.Ser483LysfsX6 | + |  | - |  | + | + | - | + | - | + | - |
| Migliore et al. [2013] | OS323 mother | F |  |  | + |  |  |  |  |  |  |  |  |  |  |
| Migliore et al. [2013] | OS329 Ⅱ-1 | M | c.1142_1285del (complex rearrangement composed of two deletions, an  inversion and a small insertion) | p.Ala381_429SerdelinsGly | + |  |  |  | - |  |  |  | - | + | - |
| Migliore et al. [2013] | OS329 Ⅱ-2 | M |  |  | + |  | + |  | - |  |  |  | - | + | - |
| Migliore et al. [2013] | OS329 Ⅱ-3 | M |  |  | + |  | + |  | - |  |  |  | - | + | - |
| Migliore et al. [2013] | OS329 sister | F |  |  |  |  |  |  |  |  |  |  |  |  |  |
| Migliore et al. [2013] | OS329 mother | F |  |  | - | - | - | - | - | - | - | - | - | - | - |
| Ji et al.[2014] | Proband | M | c.1561C>T | p.Arg521Cys | + |  | + |  | + |  |  |  | + |  |  |
| Ji et al.[2014] | Sister | F |  |  | - | - | - | - | - | - | - | - | - | - | - |
| Ji et al.[2014] | Mother | F |  |  | + |  |  |  |  |  |  |  |  |  |  |
| Ji et al.[2014] | Maternal grandmother | F |  |  | - | - | - | - | - | - | - | - | - | - | - |
