# Supplementary material for: Mid1/Mid2 expression in craniofacial development and a literature review of X‐linked opitz syndrome
Source: Mol Genet Genomic Med. 2015 Dec 12;4(1):95–105. doi: 10.1002/mgg3.183 (PMC4707030; doi:10.1002/mgg3.183)
Supplement: Supplementary file 2 — Table S2. Craniofacial features and oral cavity abnormalities in patients with MID1 gene mutations. [file MGG3-4-095-s002.doc]

**Supp. Table S2. Craniofacial Features and Oral Cavity Abnormalities in Patients with *MID1* Gene Mutations**

| Literature cases | | Sex | Impact on protein  structure | Hypertelorism | Prominent forehead | Widow's peak | Broad and/or  high nasal  bridge | Anteverted nares | CL/P | High arched palate | Broad and/or  grooved nasal tip | Flat philtrum | Ear abnormalities | Other/ comment |
| --- | --- | --- | --- | --- | --- | --- | --- | --- | --- | --- | --- | --- | --- | --- |
| Quaderi et al. [1997]  Robin et al. [1995] | OS5 Ⅳ-1 | M | p.Met438del | + |  |  |  | + | + |  |  |  |  | Thin upper lip with a smooth vermilion border. |
| Quaderi et al. [1997]  Robin et al. [1995] | OS5 Ⅳ-2 | F |  | + |  |  | + |  |  |  |  |  |  | Thin upper lip with a smooth vermilion border. |
| Quaderi et al. [1997]  Robin et al. [1995] | OS5 Ⅲ-2 | F |  | + |  |  |  |  |  |  |  |  |  |  |
| Quaderi et al. [1997]  Robin et al. [1995] | OS5 Ⅲ-4 | F |  | + |  |  |  |  |  |  |  |  |  |  |
| Quaderi et al. [1997] | OS16 two  brothers | M | p.Glu520GlyfsX18 | + |  | + | + |  | + |  |  |  |  |  |
|  |  | M |  | + |  | + | + |  | + |  |  |  |  |  |
| Quaderi et al. [1997] | OS16 mother | F |  | + |  |  |  |  |  |  |  |  |  |  |
| Quaderi et al. [1997] | OS20 | M | p.Tyr542LeuinsFIDSGRHY | + |  |  |  |  |  |  |  |  |  |  |
| Quaderi et al. [1997] | OS20 mother | F |  | + |  | + |  |  |  |  |  |  |  |  |
| Quaderi et al. [1997] | OS20 half sister | F |  | + |  | + |  |  |  |  |  |  |  |  |
| Gaudenz et al. [1998]  Brooks et al. [1992] | OS3 | M | p.Cys266Arg | + | + | + | + | + | + |  | + | + | + | Bilateral epicanthal folds; thin vermilion border of the upper lip; a prominent lower lip; micrognathia; ankyloglossia; a bifid uvula; short lingual frenulum; hypodontia. |
| Gaudenz et al. [1998]  Brooks et al. [1992] | OS3 mother | F |  | + |  |  |  | + |  | + |  |  |  | Diastemata between the mandibular anterior teeth; geographic and fissured tongue; agenesis of both maxillary third molars; a thin vermillion border of the upper lip. |
| Gaudenz et al. [1998] | OS14 | M | p.Glu317AsnfsX4 | + |  | + |  |  |  |  |  |  |  |  |
| Gaudenz et al. [1998] | OS19 two  brothers | M | r.(spl?) | + |  |  |  |  |  |  |  |  |  |  |
|  |  | M |  | + |  |  |  |  | + |  |  |  |  |  |
| Gaudenz et al. [1998] | OS19 mother | F |  | + |  |  |  |  |  |  |  |  |  |  |
| Gaudenz et al. [1998] | OS19 sister | F |  | + |  |  |  |  |  |  |  |  |  |  |
| Gaudenz et al. [1998] | OS28 | M | p.Arg495?fsX6 | + |  |  |  |  | + |  |  |  |  |  |
| Gaudenz et al. [1998] | OS28 two  females | F |  | + |  |  |  |  |  |  |  |  |  |  |
|  |  | F |  | + |  |  |  |  |  |  |  |  |  |  |
| Gaudenz et al. [1998] | OS36 | M | p.Ile536Thr | + |  | + | + |  | + |  |  |  |  |  |
| Gaudenz et al. [1998] | OS36 mother | F |  | + |  | + |  |  |  |  |  |  |  |  |

**Supp. Table S2. Continued**

| Literature cases | | Sex | Impact on protein  structure | Hypertelorism | Prominent forehead | Widow's peak | Broad and/or  high nasal  bridge | Anteverted nares | CL/P | High arched palate | Broad and/or  grooved nasal tip | Flat philtrum | Ear abnormalities | Other/ comment |
| --- | --- | --- | --- | --- | --- | --- | --- | --- | --- | --- | --- | --- | --- | --- |
| Gaudenz et al. [1998] | Patient with  sporadic OS | M | p.Glu507SerfsX4 | + |  |  |  |  |  |  |  |  |  |  |
| Cox et al. [2000] | OSP3 | M | p.Arg368X | + | + | + | + | + | + | + | - |  | + |  |
| Cox et al. [2000] | OSP5 | M | p.Leu626Pro | + | + | + | + | + | + | - | + |  | + |  |
| Cox et al. [2000] | OSP6 | M | p.Pro351LeufsX14 | + | + | + | + |  | + |  | - |  | + | Strabismus. |
| Cox et al. [2000] | OSP9 | M | p.Arg495X | + | + | + | + | + | - |  | - | + | + |  |
| Cox et al. [2000] | OSP10 | M | p.Gln468X | + | + | + | + | + | - |  | - | + | + |  |
| Cox et al. [2000] | OSP11 | M | p.Gln221_Glu252del | + | + | + | + | + | - |  | - | + | + |  |
| Cox et al. [2000] | OSP12 | M | p.Glu115X | + | + | - | + |  | + | - | - |  | + |  |
| Winter et al. [2003] | Family 1 proband | M | r.(spl?) | + |  |  |  |  | + |  |  |  |  |  |
| Winter et al. [2003] | Family 1 two  maternal cousins | M |  | + |  |  |  |  |  |  |  |  |  |  |
|  |  | M |  | + |  |  |  |  |  |  |  |  |  |  |
| Winter et al. [2003] | Family 1 maternal uncle | M |  |  |  |  |  |  | + |  |  |  |  |  |
| Winter et al. [2003] | Family 3 proband | M | r.(spl?)/p.? | + |  |  |  |  |  |  |  |  | + | Mild-down-slanting of palpebral fissures. |
| Winter et al. [2003] | Family 3 mother | F |  | + |  |  |  |  |  |  |  |  |  |  |
| De Falco et al. [2003] | O2 | M | p.Val463Phe | + | + |  | + | + | + |  | + |  | - | Cleft of epiglottis. |
| De Falco et al. [2003] | O2 mother | F |  | + |  |  | + |  |  |  |  |  |  |  |
| De Falco et al. [2003] | O5 | M | p.Gln445AlafsX31 | + |  |  |  |  | + |  |  |  |  |  |
| De Falco et al. [2003] | O9 | M | p.Arg495X | + |  |  | + | + | + |  |  |  | + | Large fontanelle; flat occiput with redundant nuchal skin; epicanthic folds. |
| De Falco et al. [2003] | O10 | M | p.Arg495X | + |  |  | + | + | - |  |  |  | - |  |
| De Falco et al. [2003] | O10 maternal  uncle | M |  | + |  |  |  |  | + |  |  |  |  | Large fontanel at birth. |
| De Falco et al. [2003] | O10 mother | F |  | + |  |  |  |  |  |  |  |  |  |  |
| De Falco et al. [2003] | O16 twins | M | r.(spl?) | + |  |  |  | + | + |  |  |  |  |  |
|  |  | M |  | + |  |  |  | + | + |  |  |  |  |  |
| De Falco et al. [2003] | O16 brother | M |  | + |  |  |  |  | + |  |  |  |  |  |
| De Falco et al. [2003] | O16 mother | F |  | + |  |  |  |  |  |  |  |  |  |  |
| De Falco et al. [2003] | O22 | M | r.(spl?) | + |  |  |  |  |  |  |  |  |  |  |
| De Falco et al. [2003] | O26 | M | p.Thr518ValfsX15 | + |  |  | + | - | - |  |  |  | - |  |
| De Falco et al. [2003] | O26 mother | F |  | + |  |  | + |  |  |  |  |  |  |  |

**Supp. Table S2. Continued**

| Literature cases | | Sex | Impact on protein  structure | Hypertelorism | Prominent forehead | Widow's peak | Broad and/or  high nasal  bridge | Anteverted nares | CL/P | High arched palate | Broad and/or  grooved nasal tip | Flat philtrum | Ear abnormalities | Other/ comment |
| --- | --- | --- | --- | --- | --- | --- | --- | --- | --- | --- | --- | --- | --- | --- |
| De Falco et al. [2003] | O34 | M | p.Cys195Phe | + |  |  | + | - | - |  |  |  | - | "Omega" shaped epiglottis. |
| De Falco et al. [2003] | O34 mother | F |  | + |  |  |  |  |  |  |  |  |  |  |
| De Falco et al. [2003] | O36 | M | p.Gln347X | + |  |  |  | - | - |  |  |  | - | Large anterior fontanel. |
| De Falco et al. [2003] | O36 brother | M |  | + |  |  |  |  |  |  |  |  |  | Open metopic suture. |
| De Falco et al. [2003] | O36 mother | F |  | + |  |  |  |  |  |  |  |  |  | Prominent metopic suture. |
| De Falco et al. [2003] | O42 | M | p.Lys370GlufsX18 | + |  |  |  |  | + |  |  |  | + | Prominent metopic suture. |
| De Falco et al. [2003] | O43 | M | p.Gly423X | + | + |  |  |  | - |  |  |  | + |  |
| Pinson et al. [2004] | Family 1 proband | M | p.Gly452Ser | + |  |  | + | - | - |  |  |  | + |  |
| Pinson et al. [2004] | Family 1 brother | M |  | + |  |  |  | - | - |  |  |  | + |  |
| Pinson et al. [2004] | Family 1 mother | F |  | + |  |  |  |  |  |  |  |  |  |  |
| Pinson et al. [2004] | Family 2 proband | M | p.Arg277X | + |  |  |  | + | + |  |  |  | + |  |
| Pinson et al. [2004] | Family 2 maternal half-brother | M |  | + |  | + | + | - | + |  |  | + | + |  |
| Pinson et al. [2004] | Patient 3 | M | r.(spl?) | + | + | + |  | + | - |  |  |  | - |  |
| Pinson et al. [2004] | Patient 3 mother | F |  | + |  |  | + |  |  |  |  |  |  | Required surgery for a short nose and a broad nasal bridge. |
| Pinson et al. [2004] | Patient 4 | M | p.Ser483LysfsX6 | + |  |  |  | + | - |  |  |  | + | Papillar coloboma. |
| Pinson et al. [2004] | Family 6 proband | M | p.Arg495X | + |  |  | + | + | + |  |  |  | + | Microcephaly; single central incisor. |
| Pinson et al. [2004] | Family 6 brother | M |  | + |  |  |  | + | - |  |  |  | ? | Microcephaly; wide anterior fontanelle. |
| Pinson et al. [2004] | Patient 9 | M | p.Val135_Cys137del | + |  |  |  | - | - |  |  |  | + |  |
| So et al. [2005] | OSF4 | M | p.Leu295Pro | + |  |  |  |  | + |  |  |  |  | Enlarged fontanelle as an infant. |
| So et al. [2005] | OSF4 brother | M |  | + |  |  |  |  |  |  |  |  |  | Large fontanelle. |
| So et al. [2005] | OSF4 mother | F |  | + |  |  |  |  |  |  |  |  |  | Lack of an incisor. |
| So et al. [2005] | OSF6 | M | p.Pro519X | + |  |  |  |  | + |  |  |  |  | Epicanthic folds. |
| So et al. [2005] | OSF6 maternal grandfather | M |  | + |  |  |  |  |  |  |  |  |  |  |
| So et al. [2005] | OSF6 mother | F |  | + |  |  |  |  |  |  |  |  |  | Epicanthic folds. |
| So et al. [2005] | OSF7 | M | p.Leu391_Cys392delinsArg | + |  |  |  |  |  |  |  |  |  | Bifid uvula; micrognathia. |
| So et al. [2005] | OSF7 mother | F |  | + |  |  |  |  |  |  |  |  |  | Short uvula; micrognathia. |
| So et al. [2005] | OSF11 | M | p.Trp552X | + |  |  |  |  | + |  |  |  |  | Squint. |
| So et al. [2005] | OSF11 mother | F |  | + |  |  |  |  |  |  |  |  |  |  |

**Supp. Table S2. Continued**

| Literature cases | | Sex | Impact on protein  structure | Hypertelorism | Prominent forehead | Widow's peak | Broad and/or  high nasal  bridge | Anteverted nares | CL/P | High arched palate | Broad and/or  grooved nasal tip | Flat philtrum | Ear abnormalities | Other/ comment |
| --- | --- | --- | --- | --- | --- | --- | --- | --- | --- | --- | --- | --- | --- | --- |
| So et al. [2005] | OSF23 | M | r.(spl?) | + |  |  | + |  | + |  | + |  | + | Short neck. |
| So et al. [2005] | OSF23 mother | F |  | + |  |  |  |  |  |  |  |  |  |  |
| So et al. [2005] | Patient 14 | M | r.(spl?) | + |  |  |  |  | + |  |  |  |  |  |
| So et al. [2005] | Patient 25 | M | p.Ser483LysfsX6 | + |  |  |  |  |  |  |  |  | + |  |
| So et al. [2005] | Patient 46 | M | p.Met438LysfsX41 | + |  |  |  |  |  |  |  |  | + | Short frenulum; bifid tip of tongue. |
| So et al. [2005] | Patient 62 | M | r.(spl?) | + |  |  |  |  | + |  |  |  | + |  |
| So et al. [2005] | Patient 75 | M | p.Arg277X | + |  |  |  |  | + |  |  |  |  |  |
| So et al. [2005] | Patient 75  maternal cousin | M |  | + |  |  |  |  |  |  |  |  |  |  |
| So et al. [2005] | Patient 75  maternal cousin | M |  | + |  |  |  |  |  |  |  |  |  |  |
| Shaw et al. [2006] | Proband | M | p.Arg495X | + |  |  |  |  | + |  |  |  |  | A neonatal mandibular incisor. |
| Shaw et al. [2006] | Brother | M |  | + |  |  |  |  |  |  |  |  |  | Two neonatal mandibular incisors; ankyloglossia. |
| Shaw et al. [2006] | Mother | F |  | + |  |  |  |  |  |  |  |  |  | Ankyloglossia. |
| Shaw et al. [2006] | Maternal grandmother | F |  | + |  |  |  |  |  |  |  |  |  | Ankyloglossia. |
| Mnayer et al. [2006] | Patient | M | p.Pro441Leu | + |  | + | + |  | - |  |  |  | + |  |
| Cho et al. [2006] | Proband | M | p.His600ProfsX12 | + |  |  | + | - | - | + |  |  | - |  |
| Cho et al. [2006] | Mother | F |  | + |  |  | + |  |  |  |  |  |  |  |
| Ferrentino et al. [2007] Fontanella et al. [2008] | OS8 | M | p.Cys145Tyr |  |  |  |  |  | + |  |  |  |  |  |
| Ferrentino et al. [2007] Fontanella et al. [2008] | OS98 | M | p.? | + |  |  |  |  | - |  |  |  |  |  |
| Ferrentino et al. [2007] Fontanella et al. [2008] | OS98 brother | M |  | + |  |  |  |  | - |  |  |  |  |  |
| Ferrentino et al. [2007] Fontanella et al. [2008] | OS112 | M | r.(spl?) | + |  |  |  |  | + |  |  |  |  |  |
| Ferrentino et al. [2007] Fontanella et al. [2008] | OS112 mother | F |  | + |  |  |  |  |  |  |  |  |  |  |
| Ferrentino et al. [2007] Fontanella et al. [2008] | OS118 | M | p.Thr133CysfsX4 | + |  |  |  |  |  |  |  |  |  |  |

**Supp. Table S2. Continued**

| Literature cases | | Sex | Impact on protein  structure | Hypertelorism | Prominent forehead | Widow's peak | Broad and/or  high nasal  bridge | Anteverted nares | CL/P | High arched palate | Broad and/or  grooved nasal tip | Flat philtrum | Ear abnormalities | Other/ comment |
| --- | --- | --- | --- | --- | --- | --- | --- | --- | --- | --- | --- | --- | --- | --- |
| Ferrentino et al. [2007] Fontanella et al. [2008] | OS118 mother | F |  | + |  |  |  |  |  |  |  |  |  |  |
| Ferrentino et al. [2007] Fontanella et al. [2008] | OS136 | M | p.Asp611Gly | + |  |  |  |  |  |  |  |  |  |  |
| Ferrentino et al. [2007] Fontanella et al. [2008] | OS136 maternal grandfather | M |  | + |  |  |  |  |  |  |  |  |  |  |
| Ferrentino et al. [2007] Fontanella et al. [2008] | OS136 maternal cousin | M |  |  |  |  |  |  | + |  |  |  |  |  |
| Ferrentino et al. [2007] Fontanella et al. [2008] | OS149 | M | p.Ile273MetfsX31 | + |  |  |  |  |  |  |  |  |  |  |
| Ferrentino et al. [2007] Fontanella et al. [2008] | OS157 mother | F |  | + |  |  |  |  |  |  |  |  |  |  |
| Ferrentino et al. [2007] Fontanella et al. [2008] | OS159 | M | p.Ala130Thr | - |  |  |  |  |  |  |  |  |  |  |
| Ferrentino et al. [2007] Fontanella et al. [2008] | OS165 mother | F |  | + |  |  |  |  |  |  |  |  |  |  |
| Ferrentino et al. [2007] Fontanella et al. [2008] | OS167 | M | p.Gln525X | + |  |  |  |  | + |  |  |  |  |  |
| Ferrentino et al. [2007] Fontanella et al. [2008] | OS167 mother | F |  | + |  |  |  |  |  |  |  |  |  |  |
| Ferrentino et al. [2007] Fontanella et al. [2008] | OS168 | M | p.? | + |  |  |  |  |  |  |  |  |  |  |
| Ferrentino et al. [2007] Fontanella et al. [2008] | OS168 maternal uncle | M |  | + |  |  |  |  |  |  |  |  |  |  |
| Ferrentino et al. [2007] Fontanella et al. [2008] | OS172 | M | p.Glu238X | + |  |  |  |  |  |  |  |  |  |  |
| Ferrentino et al. [2007] Fontanella et al. [2008] | OS172 maternal uncle | M |  | + |  |  |  |  |  |  |  |  |  |  |
| Ferrentino et al. [2007] Fontanella et al. [2008] | OS173 | M | p.Asp619del | + |  |  |  |  | + |  |  |  |  |  |
| Ferrentino et al. [2007] Fontanella et al. [2008] | OS174 | M | p.Gly653Arg | + |  |  |  |  | + |  |  |  |  |  |

**Supp. Table S2. Continued**

| Literature cases | | Sex | Impact on protein  structure | Hypertelorism | Prominent forehead | Widow's peak | Broad and/or  high nasal  bridge | Anteverted nares | CL/P | High arched palate | Broad and/or  grooved nasal tip | Flat philtrum | Ear abnormalities | Other/ comment |
| --- | --- | --- | --- | --- | --- | --- | --- | --- | --- | --- | --- | --- | --- | --- |
| Ferrentino et al. [2007] Fontanella et al. [2008] |  | M | p.? |  |  |  |  |  | + |  |  |  |  |  |
| Ferrentino et al. [2007] Fontanella et al. [2008] | OS195 | M | p.His318ThrfsX7 | + |  |  |  |  | + |  |  |  |  |  |
| Ferrentino et al. [2007] Fontanella et al. [2008] |  | M | p.? | + |  |  |  |  | + |  |  |  |  |  |
| Ferrentino et al. [2007] Fontanella et al. [2008] | OS216 | M | p.Lys370Glu |  |  |  |  |  | + |  |  |  |  |  |
| Ferrentino et al. [2007] Fontanella et al. [2008] | OS220 | M | p.Ala130Val | + |  |  |  |  | + |  |  |  |  |  |
| Ferrentino et al. [2007] Fontanella et al. [2008] | OS223 | M | p.Cys187X | + |  |  |  |  | + |  |  |  |  |  |
| Ferrentino et al. [2007] Fontanella et al. [2008] | OS227 | M | p.Ile536ValfsX3 | + |  |  |  |  |  |  |  |  |  |  |
| Ferrentino et al. [2007] Fontanella et al. [2008] | OS229 | M | p.Gly532Arg | + |  |  |  |  |  |  |  |  |  |  |
| Ferrentino et al. [2007] Fontanella et al. [2008] | OS229 brother | M |  | + |  |  |  |  |  |  |  |  |  |  |
| Ferrentino et al. [2007] Fontanella et al. [2008] | OS231 | M | p.Gln484HisfsX13 | + |  |  |  |  | + |  |  |  |  |  |
| Ferrentino et al. [2007] Fontanella et al. [2008] | OS231 mother | F |  | + |  |  |  |  |  |  |  |  |  |  |
| Ferrentino et al. [2007] Fontanella et al. [2008] | OS232 | M | p.Glu144X | + |  |  |  |  | - |  |  |  |  |  |
| Ferrentino et al. [2007] Fontanella et al. [2008] | OS235 | M | p.Cys142Ser |  |  |  |  |  | + |  |  |  |  |  |
| Fontanella et al. [2008] | OS235 maternal nephew | M |  | + |  |  |  |  |  |  |  |  |  |  |
| Fontanella et al. [2008] | Proband | M | p.Arg203GlyfsX10 | + |  |  |  |  |  |  |  |  |  |  |
| Fontanella et al. [2008] | Brother | M |  | + |  |  |  |  |  |  |  |  |  |  |
| Fontanella et al. [2008] | Patient with  sporadic OS | M | p.Pro151Leu | + |  |  |  |  |  |  |  |  |  |  |

**Supp. Table S2. Continued**

| Literature cases | | Sex | Impact on protein  structure | Hypertelorism | Prominent forehead | Widow's peak | Broad and/or  high nasal  bridge | Anteverted nares | CL/P | High arched palate | Broad and/or  grooved nasal tip | Flat philtrum | Ear abnormalities | Other/ comment |
| --- | --- | --- | --- | --- | --- | --- | --- | --- | --- | --- | --- | --- | --- | --- |
| Hsieh et al. [2008] | Patient 6 | M | p.Lys514X | + |  |  |  |  | + |  |  |  | + | Short philtrum. |
| Hu et al. [2012] | Patient | M | p.Ile568Thr | + |  |  |  |  | + |  |  |  |  | Upward slant of palpebral fissures. |
| Huning et al. [2013] | Patient | M | p.Gln221_Glu252dup | + |  | + | + |  |  | + | + |  | + | Alternated divergent strabismus; low-grade intermittent exotrophia; shallow orbits; brachycephaly; large fontanel; temporal sparse hair；large ears; mid-cleft of the tongue; short frenulum; short nose; long philtrum; small upper lip. |
| Huning et al. [2013] | Mother | F |  | + |  |  |  |  |  |  |  |  |  | Shallow orbits. |
| Migliore et al. [2013] | OS226 | M | p.? | + |  |  | + |  | - |  |  |  | + | Downslanting eyes; retrognathia; extra mandibular incisor and no wisdom teeth. |
| Migliore et al. [2013] | OS242 | M | p.? | + |  | + | + |  | + |  |  |  | + | Intermittent convergent squint and astigmatism; large anterior fontanelle; upslanting palpebral fissures. |
| Migliore et al. [2013] | OS280 | M | p.Val289SerfsX14 | + |  |  |  |  | - |  |  |  |  |  |
| Migliore et al. [2013] | OS283 | M | p.Thr149Ile | + |  |  |  |  | + |  |  |  | + | Large anterior fontanelle; glabellar haemangioma. |
| Migliore et al. [2013] | OS291 | M | p.Leu320GlufsX4 | + |  |  |  |  | + |  |  |  | - |  |
| Migliore et al. [2013] | OS300 | M | p.Cys392X | + |  |  |  |  | - |  |  |  | - |  |
| Migliore et al. [2013] | OS308 | M | p.Tyr459X | + |  | + | + |  | - | + |  |  | + | Plagiocephaly; midline scalp cutis aplasia;epicanthic folds; down slanting palpebral fissures; some missing secondary teeth; mild hearing loss. |
| Migliore et al. [2013] | OS313 | M | p.Asp123GlufsX16 | + | + |  | + |  | + |  |  |  | + |  |
| Migliore et al. [2013] | OS313 brother | M |  |  |  |  |  |  | + |  |  |  |  |  |
| Migliore et al. [2013] | OS314 | M | p.Phe617Ser | + |  | + | + |  | + |  |  |  | + |  |
| Migliore et al. [2013] | OS319 | M | p.Asn442ThrfsX38 | + |  |  |  |  | - |  |  |  |  | Turricephaly; micrognathia. |
| Migliore et al. [2013] | OS323 | M | p.Ser483LysfsX6 | + |  | + |  |  | - |  |  |  |  | Upslanting palpebral fissures; tongue-tie. |
| Migliore et al. [2013] | OS323 mother | F |  | + |  |  |  |  |  |  |  |  |  |  |
| Migliore et al. [2013] | OS329 Ⅱ-1 | M | p.Ala381_429SerdelinsGly | + |  |  |  |  |  |  |  |  |  |  |

**Supp. Table S2. Continued**

| Literature cases | | Sex | Impact on protein  structure | Hypertelorism | Prominent forehead | Widow's peak | Broad and/or  high nasal  bridge | Anteverted nares | CL/P | High arched palate | Broad and/or  grooved nasal tip | Flat philtrum | Ear abnormalities | Other/ comment |
| --- | --- | --- | --- | --- | --- | --- | --- | --- | --- | --- | --- | --- | --- | --- |
| Migliore et al. [2013] | OS329 Ⅱ-1 | M | p.Ala381_429SerdelinsGly | + |  |  |  |  |  |  |  |  |  |  |
| Migliore et al. [2013] | OS329 Ⅱ-2 | M |  | + |  |  |  |  | + |  |  |  |  |  |
| Migliore et al. [2013] | OS329 Ⅱ-3 | M |  | + |  |  |  |  | + |  |  |  |  |  |
| Ji et al.[2014] | Proband | M | p.R521C | + |  |  |  |  | + |  |  |  |  |  |
| Ji et al.[2014] | Mother | F |  | + |  |  |  |  |  |  |  |  |  | Ankyloglossia. |
