# Supplementary material for: Mid1/Mid2 expression in craniofacial development and a literature review of X‐linked opitz syndrome
Source: Mol Genet Genomic Med. 2015 Dec 12;4(1):95–105. doi: 10.1002/mgg3.183 (PMC4707030; doi:10.1002/mgg3.183)
Supplement: Supplementary file 3 — Table S3. Central nervous system defects in patients with MID1 gene mutations. [file MGG3-4-095-s003.doc]

**Supp. Table S3. Central Nervous System Defects in Patients with *MID1* Gene M**utations

| Literature cases | | Sex | Impact on protein structure | Intellectual disability/ Mental retardation/ Dev delay | Brain abnormalities |
| --- | --- | --- | --- | --- | --- |
| Quaderi et al. [1997]  Robin et al. [1995] | OS5Ⅳ-1 | M | p.Met438del | Developmental delay. | Agenesis of the corpus callosum. |
| Quaderi et al. [1997] | OS16 two brothers | M | p.Glu520GlyfsX18 | Showed mild developmental delay, especially in their speech, hyperactivity. |  |
| Quaderi et al. [1997] | OS20 | M | p.Tyr542LeuinsFIDSGRHY | Had developmental delay, functioning at a level of mild mental retardation at 3 years |  |
| Cox et al. [2000] | OSP6 | M | p.Pro351LeufsX14 | Developmental delay. | - |
| Cox et al. [2000] | OSP9 | M | p.Arg495X | Developmental delay. | Agenesis of the inferior vermis; large cisterna magna. |
| Cox et al. [2000] | OSP10 | M | p.Gln468X | Developmental delay. | Hypoplasia of the cerebellum. |
| Cox et al. [2000] | OSP11 | M | p.Gln221_Glu252del | Presented developmental delay and autistic features. | - |
| Cox et al. [2000] | OSP12 | M | p.Glu115X | Developmental delay. | - |
| De Falco et al. [2003] | O2 | M | p.Val463Phe | Mild developmental delay. |  |
| De Falco et al. [2003] | O9 | M | p.Arg495X |  | Agenesis of the corpus callosum ; hypoplasia of the vermis. |
| De Falco et al. [2003] | O16 twins | M | r.(spl?) | Have comparable mental development with only mild psychomotor retardation. | - |
|  |  |  |  |  | Hypoplasia of cerebellar vermis. |
| De Falco et al. [2003] | O22 | M | r.(spl?) | Mild mental retardation. |  |
| De Falco et al. [2003] | O26 | M | p.Thr518ValfsX15 | At the age of one year, was a sociable child with only transiently delayed motor milestones. |  |
| De Falco et al. [2003] | O34 | M | p.Cys195Phe | Developmental delay. | - |
| De Falco et al. [2003] | O36 | M | p.Gln347X | - | Dandy-Walker cyst. |
| De Falco et al. [2003] | O36 brother | M |  | - | Dandy-Walker malformation. |
| De Falco et al. [2003] | O42 | M | p.Lys370GlufsX18 | Developmental delay. |  |
| Pinson et al. [2004] | Family 2 proband | M | p.Arg277X | Developmental delay. | Hypoplasia of the vermis. |
| Pinson et al. [2004] | Family 2 maternal half-brother | M |  | Had developmental delay. Required special education. | ? |
| Pinson et al. [2004] | Family 3 proband | M | r.(spl?) | Mild mental retardation. | - |
| Pinson et al. [2004] | Family 4 proband | M | p.Ser483LysfsX6 | - | Agenesis of the inferior vermis. |
| Pinson et al. [2004] | Family 6 proband | M | p.Arg495X | Showed growth retardation, psychomotor delay with severe speech delay at 4 1/2 years of age. | Microcephaly; hypoplasia of the vermis. |
| Pinson et al. [2004] | Family 6 brother | M |  | Growth retardation. | Microcephaly; hypoplasia of the vermis; hypoplasia of the posterior corpus callosum. |
| So et al. [2005] | OSF23 | M | r.(spl?) | Global developmental delay; speech delay. |  |
| So et al. [2005] | Patient 25 | M | p.Ser483LysfsX6 | Showed developmental delay at ages 4 and 16 months. |  |
| So et al. [2005] | Patient 46 | M | p.Met438LysfsX41 | Showed mild mental retardation at age 9 years. |  |
| Shaw et al. [2006] | Proband | M | p.Arg495X | Had delay in speech development at 2 years. |  |
| Ferrentino et al. [2007] Fontanella et al. [2008] | OS98 | M | p.? | Mild. | - |

**Supp. Table S3. Continued**

| Literature cases | | Sex | Impact on protein structure | Intellectual disability/ Mental retardation/ Dev delay | Brain abnormalities |
| --- | --- | --- | --- | --- | --- |
| Ferrentino et al. [2007] Fontanella et al. [2008] | OS149 | M | p.Ile273MetfsX31 | - | + |
| Ferrentino et al. [2007] Fontanella et al. [2008] | OS229 | M | p.Gly532Arg | + |  |
| Ferrentino et al. [2007] Fontanella et al. [2008] | OS229 brother | M |  | Mild. |  |
| Ferrentino et al. [2007] Fontanella et al. [2008] | OS231 | M | p.Gln484HisfsX13 |  | + |
| Fontanella et al. [2008] | Proband | M | p.Arg203GlyfsX10 | + |  |
| Fontanella et al. [2008] | Patient with  sporadic OS | M | p.Pro151Leu | + |  |
| Hsieh et al. [2008] | Patient 6 | M | p.Lys514X | Reached all motor developmental milestones on time and smiled at 6 weeks, held his head up at 2–3 months, turned over at 4 months, sat at 6.5 months, crawled at 7 months, and walked at 11 months. Showed speech delayed with his first word at 15 months and first two-word combination phrase at 30–36 months. His speech pattern exhibits echolalia. Had autistic tendencies and behavior problems. Had global developmental delay and attended 11th grade at age 17. |  |
| Hu et al. [2012] | Patient | M | p.Ile568Thr |  | Hypoplasia of the corpus callosum; hypoplasia of the cerebellum. |
| Huning et al. [2013] | Patient | M | p.Gln221_Glu252dup | At the age of two years and three months, was within the normal range for his age for growth parameters and development. Walks at the age of 11 months, had good comprehensive skills since the age of one year but limited speech, three words at the age of 1 year 11 months and the first sentence at the age of two years and two months. | Brachycephaly. |
| Migliore et al. [2013] | OS226 | M | p.? |  | Slight underdevelopment of the cerebellum. |
| Migliore et al. [2013] | OS283 | M | p.Thr149Ile | At the age of 4, had some speech and language delay, using 2-3 word sentences, was within the normal range for his age for some skills such as performance and practical reasoning, but his locomotor and personal social skills were noted to be poor, with lack of confidence. | - |
| Migliore et al. [2013] | OS291 | M | p.Leu320GlufsX4 | -  Academically he has been successful and is attending university. He has always struggled, however, more than his siblings academically, and socially things have been difficult for him. |  |
| Migliore et al. [2013] | OS300 | M | p.Cys392X |  | Blake's cyst in the posterior cranial fossa with upper-rostral position of the cerebellum. |
| Migliore et al. [2013] | OS308 | M | p.Tyr459X | Has autistic spectrum disorder. | Plagiocephaly. |

**Supp. Table S3. Continued**

| Literature cases | | Sex | Impact on protein structure | Intellectual disability/ Mental retardation/ Dev delay | Brain abnormalities |
| --- | --- | --- | --- | --- | --- |
| Migliore et al. [2013] | OS314 | M | p.Phe617Ser | - | Hypoplasia of cerebellar vermis and cisterna magna; hypoplasia of the splenium of the corpus callosum. |
| Migliore et al. [2013] | OS323 | M | p.Ser483LysfsX6 | - | Enlargement of the cisterna magna and Ⅵ ventricle. |
| Migliore et al. [2013] | OS329 Ⅱ-1 | M | p.Ala381_429SerdelinsGly | He has some difficulty at school. |  |
