# Supplementary material for: Mid1/Mid2 expression in craniofacial development and a literature review of X‐linked opitz syndrome
Source: Mol Genet Genomic Med. 2015 Dec 12;4(1):95–105. doi: 10.1002/mgg3.183 (PMC4707030; doi:10.1002/mgg3.183)
Supplement: Supplementary file 4 — Table S4. Heart defects in patients with MID1 gene mutations. [file MGG3-4-095-s004.doc]

**Supp. Table S4. Heart Defects in Patients with *MID1*** Gene Mutations

| Literature cases | | Sex | Impact on protein structure | Atrial septal defect | Ventricular septal defect | Patent ductus arteriosus | Other/ comment |
| --- | --- | --- | --- | --- | --- | --- | --- |
| Gaudenz et al. [1998] | OS36 | M | p.Ile536Thr |  | + |  |  |
| Cox et al. [2000] | OSP5 | M | p.Leu626Pro | + |  | + |  |
| Cox et al. [2000] | OSP6 | M | p.Pro351LeufsX14 | + | + |  | Coarctation of the aorta; other cardiac anormalies. |
| Cox et al. [2000] | OSP9 | M | p.Arg495X |  | + |  |  |
| Cox et al. [2000] | OSP10 | M | p.Gln468X |  | + |  | Pulmonary stenosis. |
| Winter et al. [2003] | Family 3 proband | M | r.(spl?)/p.? | + |  | + | Coarctation of the aorta; abnormal venous return. |
| De Falco et al. [2003] | O16 twins | M | r.(spl?) |  |  |  | Persistent superior vena cava |
|  |  | M |  |  | + |  |  |
| De Falco et al. [2003] | O16 brother | M |  |  |  | + |  |
| De Falco et al. [2003] | O22 | M | r.(spl?) |  |  |  | Had cardiac problems (unspecified). |
| De Falco et al. [2003] | O36 | M | p.Gln347X |  |  |  | Bilateral superior vena cava; patent foramen ovale. |
| De Falco et al. [2003] | O42 | M | p.Lys370GlufsX18 |  |  | + |  |
| Pinson et al. [2004] | Patient 3 | M | r.(spl?) |  |  |  | Had heart defects (unspecified). |
| Pinson et al. [2004] | Family 6 brother | M |  |  | + |  |  |
| So et al. [2005] | OSF6 | M | p.Pro519X | + |  |  |  |
| Shaw et al. [2006] | Proband | M | p.Arg495X |  |  |  | Had bilateral superior vena cavae, but a structurally normal heart. |
| Ferrentino et al. [2007] Fontanella et al. [2008] | OS168 | M | p.? |  |  |  | Had heart defects (unspecified). |
| Ferrentino et al. [2007] Fontanella et al. [2008] | OS168 maternal uncle | M |  |  |  |  | Had heart defects (unspecified). |
| Ferrentino et al. [2007] Fontanella et al. [2008] | OS173 | M | p.Asp619del |  |  |  | Had heart defects (unspecified). |
| Ferrentino et al. [2007] Fontanella et al. [2008] | OS229 brother | M |  |  |  |  | Had heart defects (unspecified). |
| Ferrentino et al. [2007] Fontanella et al. [2008] | OS231 | M | p.Gln484HisfsX13 |  |  |  | Had heart defects (unspecified). |
| Fontanella et al. [2008] | OS235 maternal nephew | M |  |  |  |  | Had heart defects (unspecified). |
| Fontanella et al. [2008] | Brother | M |  |  |  |  | Had heart defects (unspecified). |
| Migliore et al. [2013] | OS300 | M | p.Cys392X |  |  |  | Agenesis of the venous duct |
| Migliore et al. [2013] | OS314 | M | p.Phe617Ser |  |  |  | Patent foramen ovale |
| Migliore et al. [2013] | OS319 | M | p.Asn442ThrfsX38 | + |  | + |  |
| Ji et al.[2014] | Proband | M | p.R521C | + |  |  |  |
