# Supplementary material for: Mid1/Mid2 expression in craniofacial development and a literature review of X‐linked opitz syndrome
Source: Mol Genet Genomic Med. 2015 Dec 12;4(1):95–105. doi: 10.1002/mgg3.183 (PMC4707030; doi:10.1002/mgg3.183)
Supplement: Supplementary file 5 — Table S5. Clinical findings in patients with MID2 gene mutation. [file MGG3-4-095-s005.docx]

**Supp. Table S5. Clinical Findings in Patients with *MID2* Gene Mutation**

| Literature cases | | Sex | Genetic findings | Impact on  protein structure | Craniofacial abnormalities | | | | |  | Intellectual disability/ MR/ Dev delay | | | | | | Brain abnormalities |
| --- | --- | --- | --- | --- | --- | --- | --- | --- | --- | --- | --- | --- | --- | --- | --- | --- | --- |
|  |  |  |  |  | Long face | Large ears | Short philtrum | Strabismus/squint | Other/ comment |  | Impaired cognition | Mental Retardation | Unclear speech | Hyperactivity | Destructive behavior | Seizures |  |
| Geetha et al. | Ⅲ6 | M | c.1040G>A | p.Arg347Gln | + | - | - | + |  |  | + | Mild | + | +* | - | + | Gliotic area,frontal lobe;  bifrontal atrophy |
|  | Ⅲ22 | M |  |  | - | - | + | + |  |  | + | Mild | + | +* | - | - | Bilateral white matter  hypomylination in  periventricular area |
|  | Ⅲ23 | M |  |  | + | + | - | + |  |  | + | Mild | + | +* | - | - | - |
|  | Ⅳ2 | M |  |  | + | - | - | - |  |  | + | Mild-moderate | - | + | - | - | - |
|  | Ⅳ4 | M |  |  | + | + | - | + | Ptosis ; optic  atrophy left eye |  | + | Severe | + | +** | + | Two or three times | - |
|  | Ⅳ15 | M |  |  | - | - | - | - |  |  | + | Severe | Makes abnormal sounds | +** | + | Two or three times | - |

+* Occasional uncontrolable aggression and screaming episodes

+** Very hyperactive and requires constant attention
